# G-protein Signaling Components GCR1 and GPA1 Mediate Responses to Multiple Abiotic Stresses in *Arabidopsis*

**DOI:** 10.3389/fpls.2015.01000

**Published:** 2015-11-18

**Authors:** Navjyoti Chakraborty, Navneet Singh, Kanwaljeet Kaur, Nandula Raghuram

**Affiliations:** University School of Biotechnology, Guru Gobind Singh Indraprastha UniversityDwarka, New Delhi, India

**Keywords:** *Arabidopsis*, G-protein, GPA1, GCR1, abiotic stress, enzyme assays, qPCR

## Abstract

G-protein signaling components have been implicated in some individual stress responses in *Arabidopsis*, but have not been comprehensively evaluated at the genetic and biochemical level. Stress emerged as the largest functional category in our whole transcriptome analyses of knock-out mutants of GCR1 and/or GPA1 in *Arabidopsis* (Chakraborty et al., 2015a,b). This led us to ask whether G-protein signaling components offer converging points in the plant's response to multiple abiotic stresses. In order to test this hypothesis, we carried out detailed analysis of the abiotic stress category in the present study, which revealed 144 differentially expressed genes (DEGs), spanning a wide range of abiotic stresses, including heat, cold, salt, light stress etc. Only 10 of these DEGs are shared by all the three mutants, while the single mutants (GCR1/GPA1) shared more DEGs between themselves than with the double mutant (GCR1-GPA1). RT-qPCR validation of 28 of these genes spanning different stresses revealed identical regulation of the DEGs shared between the mutants. We also validated the effects of cold, heat and salt stresses in all the 3 mutants and WT on % germination, root and shoot length, relative water content, proline content, lipid peroxidation and activities of catalase, ascorbate peroxidase and superoxide dismutase. All the 3 mutants showed evidence of stress tolerance, especially to cold, followed by heat and salt, in terms of all the above parameters. This clearly shows the role of GCR1 and GPA1 in mediating the plant's response to multiple abiotic stresses for the first time, especially cold, heat and salt stresses. This also implies a role for classical G-protein signaling pathways in stress sensitivity in the normal plants of Arabidopsis. This is also the first genetic and biochemical evidence of abiotic stress tolerance rendered by knock-out mutation of GCR1 and/or GPA1. This suggests that G-protein signaling pathway could offer novel common targets for the development of tolerance/resistance to multiple abiotic stresses.

## Introduction

Plants encounter a variety of abiotic and biotic environmental stresses, which result in substantial loss in yield of crops worldwide. Greenhouse gas emissions and climate change could further exacerbate various stresses that plants have to encounter (Challinor et al., 2014). The abiotic stresses include temperature variations (both low and high), flood, drought, and salinity. The molecular mechanisms of stress response have been extensively researched and reviewed (Cabello et al., 2014; Suzuki et al., 2014; Tanveer et al., 2014; Parihar et al., 2015). However, the search for new genetic targets for crop improvement toward stress tolerance is far from complete. Signaling mechanisms in plant stress response are of particular interest in this regard, as their extensive cross talk in plants could reveal common genetic targets to deal with multiple stresses. The signaling mechanisms in plants during low and high temperature, drought, and salinity are different and yet related to each other (Nakashima et al., 2014; Smékalová et al., 2014). Each of these stresses provide different cues and elicit changes from the plant at different levels, including physiological and biochemical levels, as well as at the level of gene expression. The physiological changes are easily measurable in terms of germination, root length, shoot length, etc., whereas the biochemical changes are measured by markers such as lipid peroxidation, SOD assay, APx assay, etc. (Cabello et al., 2014).

G-proteins and GPCR have been associated with several stress-signaling pathways in plants (Pandey et al., 2015). G-proteins transmit the signal through downstream effectors like ion channels, phospholipases, kinases/phosphatases and other GTPases (Xu et al., 2015). G-proteins regulate the activity of many enzymes like phosphatidylinositol-phospholipase C (PLC) and phospholipase D (PLD) (Apone et al., 2003). They in turn modulate the expression of stress-responsive genes like LEA and LEA-like genes under different stress conditions in *Arabidopsis* (Zhao, 2015), clearly indicating the involvement of G-proteins in stress signaling. Similarly, the loss-of-function mutant of GCR1 in *Arabidopsis* was reported to be resistant to drought stress and also showed higher expression levels of few known drought- and ABA-regulated genes (Pandey and Assmann, 2004). This was also consistent with their finding that GCR1 acts as a negative regulator of GPA1-mediated ABA responses in *Arabidopsis* guard cells. In tobacco, transgenic lines overexpressing Gα and Gβ from pea revealed the role of Gα in salinity and high temperature stress response, while Gβ was linked to heat tolerance (Misra et al., 2007). Though, most of the stress-related studies on GPA1 have been done on ABA and biotic stresses (Alvarez et al., 2011; Wang et al., 2011; Urano et al., 2013), recent studies in *Arabidopsis* revealed that G-proteins are also involved in growth under salt stress (Colaneri et al., 2014), as well as cellular senescence and cell division in rice and maize (Urano et al., 2014).

Though important, these scattered findings were not enough to suggest widespread role of G-protein signaling components in transducing multiple stress signals. Recently, our whole transcriptome analyses of loss-of-function mutants of GCR1 and GPA1 in *Arabidopsis* revealed stress response pathways as the largest functional cluster of differentially expressed genes (Chakraborty et al., 2015a,b). Our analysis of the GCR1-GPA1 double mutant further confirmed the higher functional overlap of stress response as a category at the process level, despite the limited overlap between the mutants in terms of the DEGs themselves (Chakraborty et al., submitted). These results led us to ask whether G-protein signaling components offer converging points in the plant's response to multiple abiotic stresses (especially GCR1 and GPA1) in *Arabidopsis*. The present paper tested this hypothesis by a thoroughly detailed analysis of the abiotic stress-related category of DEGs revealed in our functional genomic analyses of the single and double mutants, as well as by validating them parallelly under three stresses *viz*., cold, heat, and salt.

## Materials and methods

### *In-silico* analysis of stress responsive genes

For this study, we used our transcriptome data obtained from the single and double mutants of GPA1 and GCR1 (GSE 40217). The list of DEGs from the transcriptome of each of the mutants was used separately as input data to generate an abiotic stress responsive dataset for each of the mutant by comparing against the stress responsive transcription factor database (STIFDB2.0). These abiotic stress responsive genes were then subjected to Venn selection to check their overlap in the mutants. Further each of these gene list were put as an input to expression browser against abiotic stress series of AtGenexpress (Toufighi et al., 2005) as background data to check their expression profile in previous expression data.

### Plant material and stress treatments

*Arabidopsis thaliana* wild type, Ws2 and knock-out mutants devoid of either GPA1 (*gpa1-5*) or GCR1 (*gcr1-5*) or both (*gpa1-5gcr1-5*) were grown on 1X B5 medium hydroponically in a growth chamber at 22 ± 1°C with a light intensity of 150 μM s^−1^ m^−2^ and a photoperiod of 16:8 h of light:dark cycle. The seeds were vernalized prior to inoculation at 4°C for 2–3 days. The seedlings were allowed to grow for 10 days followed by stress treatments. For cold stress, the seedlings were placed at 4°C for overnight (Al-Quraan et al., 2012); heat stress was given at 37°C for 4 h (Barah et al., 2013); and salt stress was given using 100 mM NaCl for 12 h (Colaneri et al., 2014). RWC was performed immediately after stress treatments. Rest of the tissues of the control as well as stressed plants were harvested in liquid nitrogen and kept at −80°C till further use. For germination studies, vernalized seeds were inoculated onto 1X B5 plates solidified using 0.4% ClariGel (Hi-Media, India) and incubated at the appropriate temperatures. For salt stress, seeds were plated on 1X B5 plates containing 100 mM NaCl and incubated at 22 ± 1°C. We used 5 plates for each of the conditions. The plates were scored for germination after 3 days. For, measurement of root and shoot length under stress and control conditions, seeds were placed on similar kind of plates as given above and incubated in vertical position at appropriate conditions. The root and shoot lengths were measured after a week.

### RT-qPCR validation of stress responsive genes

In order to validate the stress response of the wild type and the three mutants, 28 DEGs were picked from all the mutants in such a way that some of them were common to at least two of the three mutants and some were unique to any one of the three mutants. This resulted in 16, 13, and 20 DEGs picked from gcr1-5, gpa1-5, and gpa1-5gcr1-5, respectively, including some well-characterized stress-responsive genes like RD29A, RD26, ERF13, CML38, etc. Their sequences were obtained from TAIR and primers were designed using PrimerQuest tool of IDT. Total RNAs were isolated from the control and stressed tissues and the RNA samples were analyzed using spectrophotometer and electrophoresis to determine the quantity and quality. The total RNAs were used for qPCR with gene-specific primers. GPA1 and/or GCR1 responsive DEGs were verified by RT-qPCR using the instrument Stratagene Mx3000P (Agilent technologies) using standard conditions. Typically, total RNA was digested by RNase free DNase (Fermantas), repurified, quantified, and 5 μg of RNA was used for cDNA preparation for each biological replicate using Oligo(dT) primers and RevertAid reverse transcriptase (Fermentas). Sequences for designing the primers were obtained from TAIR. PCR amplifications were performed in 20 μl by using the BrilliantIII Ultrafast SYBR Green QPCR mastermix (Agilent Technologies) with 1.0 μl of sample cDNA and 100 n moles of each gene-specific primer. Primer efficiency was determined by serial dilution of the template and only primers that worked at 90–110% efficiency were used for all qPCR analyses (Supplementary Table S1). The specificity of primer pairs was obtained by melting curve analysis of the amplicons. Actin2 (ACT2) was used as an internal control for normalization. Quantification of the relative changes in gene expression was performed by using the 2–11 CT method (Pfaffl, 2001).

### Relative water content (RWC)

Relative water content of the mutants and the wild type were measured (Slavík, 1974) after the control and stress treatments. A seedling was removed and weighed (W). The seedling was then floated on de-ionized water in a Petridish/pre-weighed vial and kept at 10°C for 4 h. The seedling was then removed and wiped the surface water using a paper towel. This surface dried seedling was weighed again (TW). The seedling was then kept for drying in an 80 °C hot air oven overnight/for 24 h. The dried seedling was weighed again (DW) and RWC was calculated using the below mentioned formula:

$$\begin{array}{l} RWC\left(\%\right)=\frac{W-DW}{TW-DW}\;\times 100 \end{array}$$

### Proline content

Proline was extracted by heating the tissue (250 mg) twice in 80% ethanol and once with 50% ethanol, to obtain the final extract in a 70:30 mixture of ethanol and water. Proline standards (0.04–1 mM) were prepared by dissolving standard proline in 70:30 ethanol:water mixture. 50 μl of extract/standard was added to 100 μl of reaction mixture containing 1.0% (w/v) ninhyrin in 60% acetic acid and 20% (v/v) ethanol (Reaction mixture must be protected from light). Then the tubes were sealed and heated at 95°C for 20 min and then allowed to cool to room temperature. The mixture was then centrifuged at 25,000 rpm for 1 min. One hundred microliter of this mix was then transferred to a microplate well and absorbance was taken at 520 nm (Bates et al., 1973). The proline content was estimated against the standard curve generated.

### Malondialdehyde assay (MDA)/lipid peroxidation assay

Plant tissue (0.1 g) was crushed to fine powder in liquid nitrogen, added to 3 ml of 10% TCA and mixed well. The tube was then centrifuged at 12,000 rpm for 20 min. 2 ml of supernatant was taken and 2 ml solution of 10% TCA containing 0.6% TBA was added to it. The mixture was then heated at 85°C for 30 min and allowed to cool to room temperature. Absorbance was then taken at 450, 532, and 600 nm. MDA content was calculated using the formula (Hodges et al., 1999):

$$\begin{array}{l} MDA\;content=\left(Z\times 6.45\right)-\left(A_{450}\times 0.56\right)\mu \text{M}∕\text{gFw} \end{array}$$

where, *Z* = (*A*_532_ − *A*_600_), gFw = Fresh weight (in g)

### Catalase assay

Plant tissue (0.25 g) was crushed using liquid nitrogen to fine powder and added to 1 ml 0.1% (w/v) TCA in an ice-bath. The mixture was then centrifuged at 12,000 g for 15 min at 4°C and the supernatant (100 μl) was taken. To it, 50 μl of 10 mM potassium phosphate (pH 7.0) and 100 μl 1 M potassium iodide were added, vortexed and absorbance was measured at 390 nm (Velikova et al., 2000). Final calculation was done against the standard curve generated using commercial hydrogen peroxide.

### Ascorbate peroxidase (APx) estimation

Tissue (0.25 g) was ground to a fine powder with liquid nitrogen and added to 1 ml extraction buffer containing 50 mM sodium phosphate (pH 7.5), 1 mM PEG, 1 mM PMSF, 8% (w/v) PVPP, and 0.01% (v/v) Triton X-100. The mix was centrifuged at 18,000 rpm for 20 min and the supernatant was transferred to a fresh tube. The extract (20 μl) was added to 1 ml of reaction mixture (0.2 M Tris-Cl, pH 7.8; 0.25 mM ascorbic acid, and 0.5 mM H_2_O_2_), mixed by inversion and absorbance recorded after 10 min at 290 nm till the absorbance stabilized (Nakano and Asada, 1981). The enzyme activity was calculated as follows:

$$\begin{array}{l} APx\;activity=\frac{A_{2}-A_{1}}{T_{2}-T_{1}}\mathrm{per}\;\mathrm{mg}\;\mathrm{protein} \end{array}$$

### Superoxide dismutase (SOD) assay

The tissue (0.25 g) was homogenized in 100 mM TEA buffer (pH 7.4), centrifuged at 16,000 rpm for 20 min and the supernatant was used as the crude extract. The assay mixture was prepared by adding 10 mM TEA buffer (pH 7.4), 7.5 mM NADH, 100 mM/50 mM EDTA/MnCl_2_, 10 mM 2-mercaptaethanol and the crude extract. Decrease in absorbance was monitored at 340 mM for 15 min (Beauchamp and Fridovich, 1971). The enzyme activity was calculated as:

$$\begin{array}{l} SOD\;activity=\frac{A_{2}-A_{1}}{T_{2}-T_{1}}\mathrm{per}\;\mathrm{mg}\;\mathrm{protein} \end{array}$$

### Statistical analyses

The data were analyzed statistically using ANOVA (analysis of variance) and the differences among the mean values were compared with Duncan's Multiple Range Test (DMRT) (*P* < 0.05) using Sigmaplot ver. 11 (Wass, 2009). All the results were expressed as mean ± SD of three independent experiments.

## Results

### *In-silico* analysis GPA1/GCR1-responsive genes in abiotic stress

**Figure 1:**
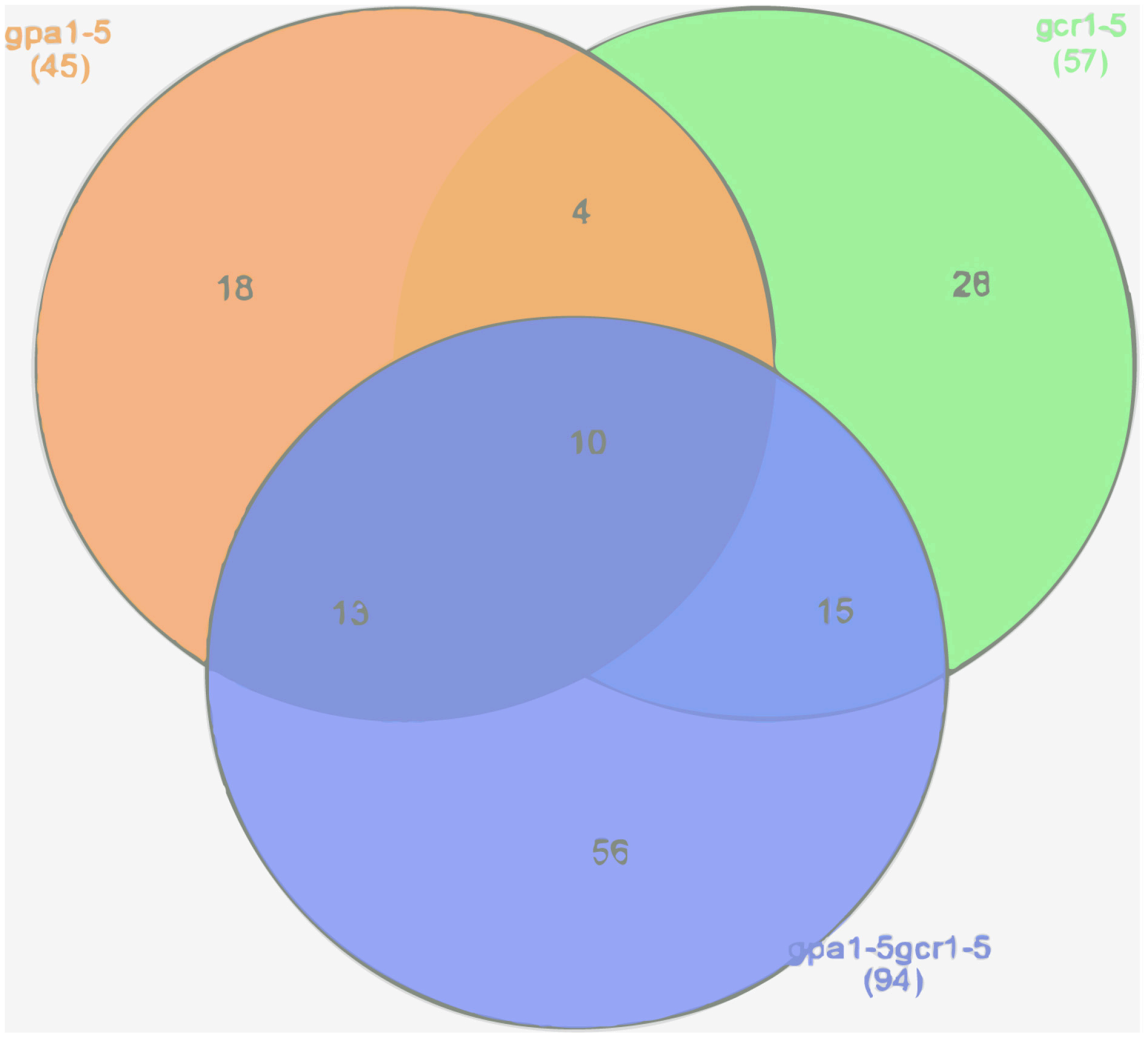
**Venn selection of abiotic stress responsive DEGs in GPA1/GCR1 mutants**. DEGs from the transcriptome of each of the above mutant (GEO accession no. GSE 40217) was compared to abiotic stress responsive genes from STIFDB2.0 to obtain GPA1/GCR1 regulated stress responsive genes. Their distribution in the mutants was checked using Venn selection.

In this study, our comparison of DEGs from GPA1/GCR1-responsive transcriptomes to the known list of abiotic stress-responsive genes (STIFDB2.0) (Naika et al., 2013) revealed 57 DEGs (49 up/8 down) from the *gcr1-5 mutant*, 45 (30 up/15 down) from the *gpa1-5 mutant*, and 94 (68 up/26 down) from the *gpa1-5gcr1-5* double mutant (Supplementary Table S1), relative to the wild type (Ws2) in each case. When these stress responsive gene lists obtained from each of the mutant was compared to each other, we found that 10 DEGs are shared by all the three mutants, while 4 additional genes were only shared between the two single mutants. Interestingly, each of the single mutants share many more DEGs with the double mutant, with 15 of them from *gcr1-5* and 13 from the *gpa1-5* (Figure 1). All these DEGs span a wide range of abiotic stresses, including heat, cold, salt, light stress etc. (Supplementary Table S2). Further, when each of the abiotic stress responsive gene lists from the mutants were subjected to Expression Browser tool of Bio Array Resource, the expression value of each of the genes under different abiotic stresses like cold, oxidative, salt, heat, etc. was shown as a heatmap (Supplementary Figures S1–S3). We found that each of the genes in the input list not only showed different fold change value under different stress conditions but also varied based on the duration of the treatment given (0.5–24 h) under each stress.

### qPCR validation of GPA1/GCR1-responsive genes in abiotic stress

**Figure 2:**
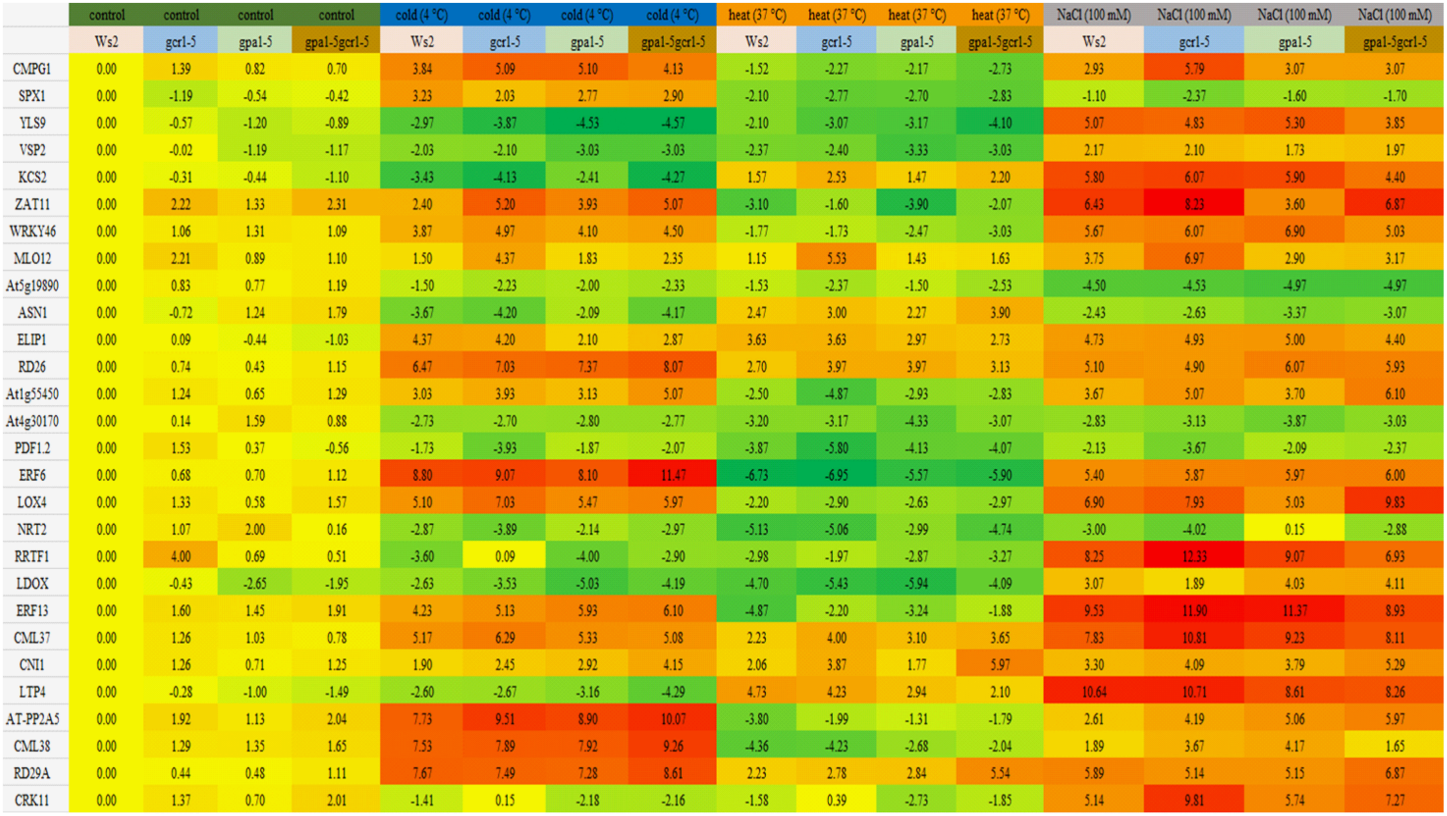
**qPCR of stress responsive genes validate the role GPA1 and GCR1 in regulating abiotic stresses**. These genes have been implicated in various abiotic stress response previously and also found to be differentially regulated in our transcriptome data (GEO accession no. GSE 40217). The values are average log2 fold change values obtained from 3 independent experiments each having technical triplicates (The final values as log2 fold change ±SE and statistical significance is given as Supplementary Table). Red represents up-regulation; green represents down-regulation; yellow is non-differential. The intensity of color represents the level of differential regulation.

Most of the genes were down-regulated under heat stress in Ws2 as well as all the mutants. Both wild- type and all 3 mutants behaved similarly in terms of up/down regulation of genes under stress conditions, though the extent of such regulation varied occasionally. The extent of regulation was much higher in the stress treatment conditions than in the control in all the plants (Figure 2). Out of all the 28 genes validated, only 4 were found to be down-regulated and only 5 were found to be up-regulated in all the conditions in both the wild-type and the mutants. Under cold stress, 7 genes were found to be highly up-regulated and 6 genes were highly down-regulated in all. The up-regulated genes include well-known stress responsive genes like AT-PP2A5, ERF6, CML38, RD29A, and RD26; while the down-regulated ones include YLS9, VSP2, RRTF1, and a peroxidase family protein. Only 9 genes were found to be up-regulated under heat stress in all the plants and the rest were down-regulated. The up-regulated genes include MLO12, ELIP1, RD29A, RD26, ASN1 etc. The highly down-regulated genes included ERF6 and 13, PDF1.2, ZAT11, LDOX, peroxidase family protein, etc. Most of the genes were found to be up-regulated under salt stress with only 6 genes being down-regulated. The up-regulated ones included ERF13, CML37, RRTF1, etc. while the down-regulated ones included NRT2.1, SPX1, PDF1.2, ASN1, and two members of peroxidase family. The final fold change values of each of the selected genes with standard error and statistical significance is given in Supplementary Table S3.

### Phenotypic validation of tolerance to different abiotic stresses in all three mutants

**Figure 3:**
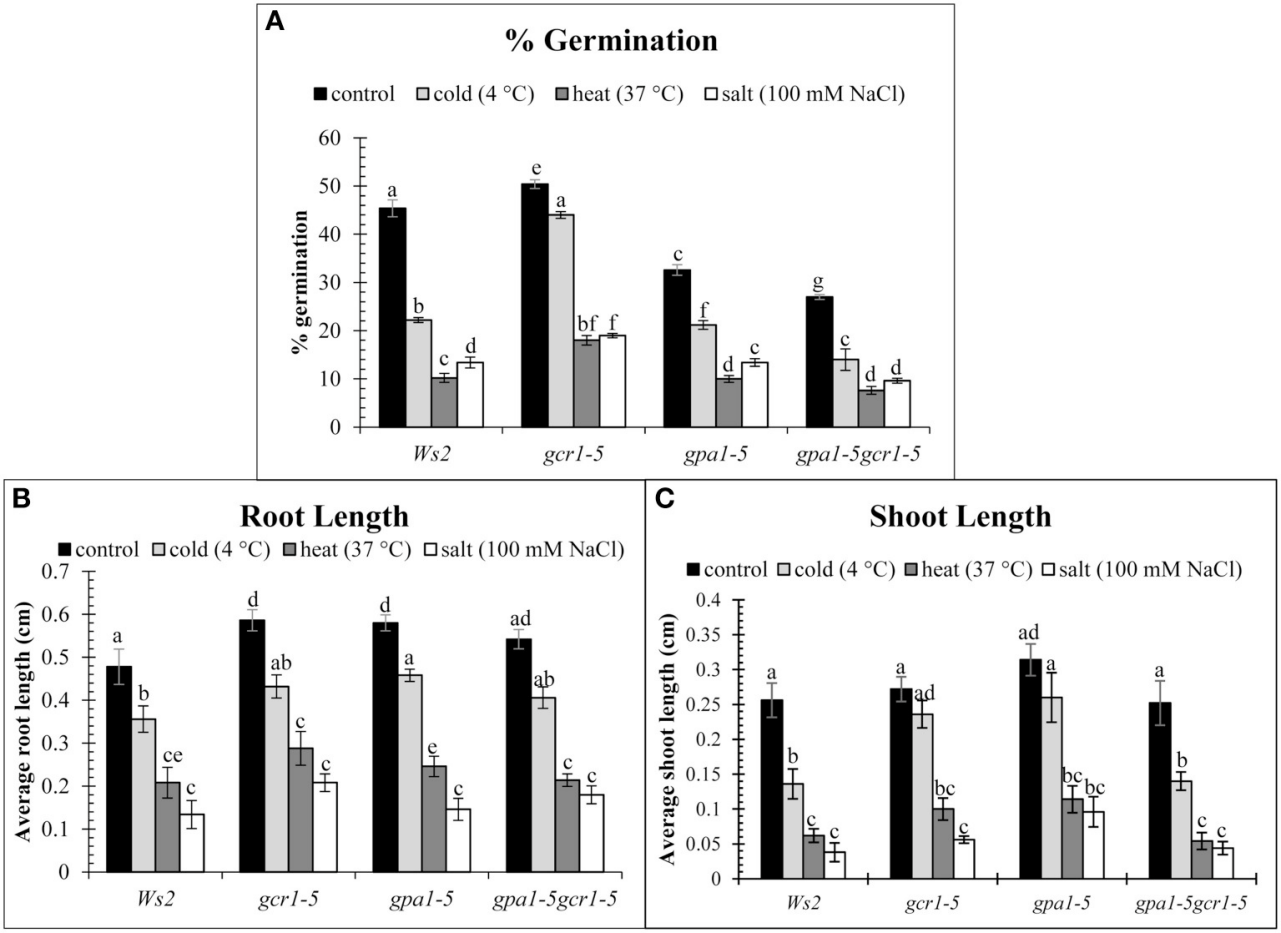
**Phenotypic characterization of mutants under control and stress conditions**. Germination (%) **(A)**, Average root length **(B)** and Average shoot length **(C)**. The values are mean of 3 independent experiments. Each reading was average of atleast 10 independent plants/plates. Data is represented as mean ± SE. Values followed by different letters are significantly different at 5% level as determined by Duncan's test.

The phenotypes of the wild-type (Ws2) and the mutants (*gcr1-5, gpa1-5*, and *gpa1-5gcr1-5*) under control and stress conditions were measured in terms of % germination, root and shoot length. We found that under control conditions, *gcr1-5* showed better germination (~50%) than Ws2 (~45%) while the other two mutants had lower germination rate (~33% and 27%) than Ws2 (Figure 3A). Germination percentage reduced drastically in the heat and salt stressed seeds of all, though the mutants had slightly higher germination percentages. *gcr1-5* showed better germination under cold stress than any other mutant and wild-type. Germination under stressed conditions was lowest in the double mutant (*gpa1-5gcr1-5*) while *gpa1-5* showed germination level similar to the wild type under salt and heat stress. Reduction in root length of both wild type and the mutants were observed when grown under stress conditions but the change was almost similar in them (Figure 3B). When shoot lengths of the wild-type and the mutants under control and stress treatments were compared, we found that the shoot length were almost comparable in Ws2 and the mutants, but the effect of heat and salt were more severe in all. The single mutants (*gpa1-5* and *gcr1-5*) showed better shoot length under cold conditions than the double mutant (Figure 3C).

### Validation of abiotic stress tolerance by non-enzymatic stress markers

**Figure 4:**
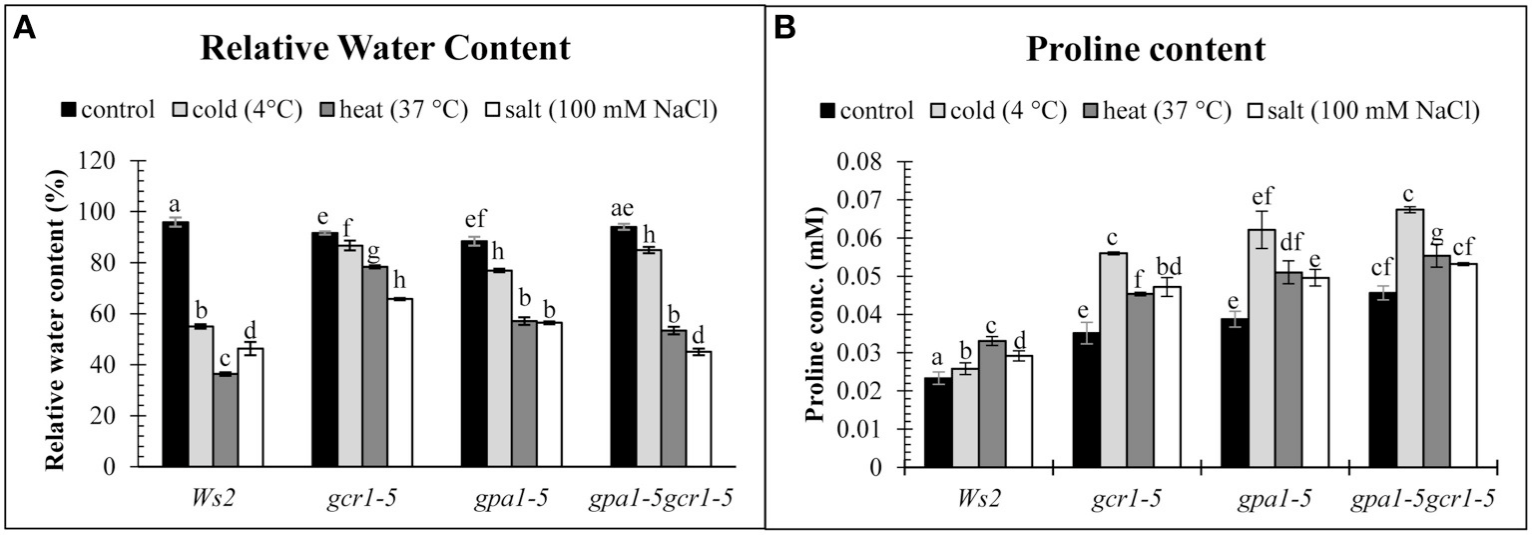
**Non-enzymatic stress markers, RWC (A) and Proline content (B) validate the roles of GCR1/GPA1 in abiotic stress**. The values are mean of 3 independent experiments each having technical triplicates. Data is represented as mean ± SE. Values followed by different letters are significantly different at 5% level as determined by Duncan's test.

When treated with different stresses in parallel, the RWC in the wildtype (Ws2) decreased to 55, 36, and 46% in cold, heat and salt stress respectively, while the mutants showed higher RWC under the same conditions. The mutant RWC values under cold, heat and salt stress were found to be 87, 78, and 66% respectively in *gcr1-5*, 77, 57, and 56% in *gpa1-5* and 85, 53, and 45% in the double mutant *gpa1-5gcr1-5* (Figure 4A). Similarly, we found that proline content was much higher in all the three mutants relative to WT under all three stresses, with maximum proline accumulation under cold stress (Figure 4B).

### Validation of abiotic stress tolerance by enzymatic stress markers

**Figure 5:**
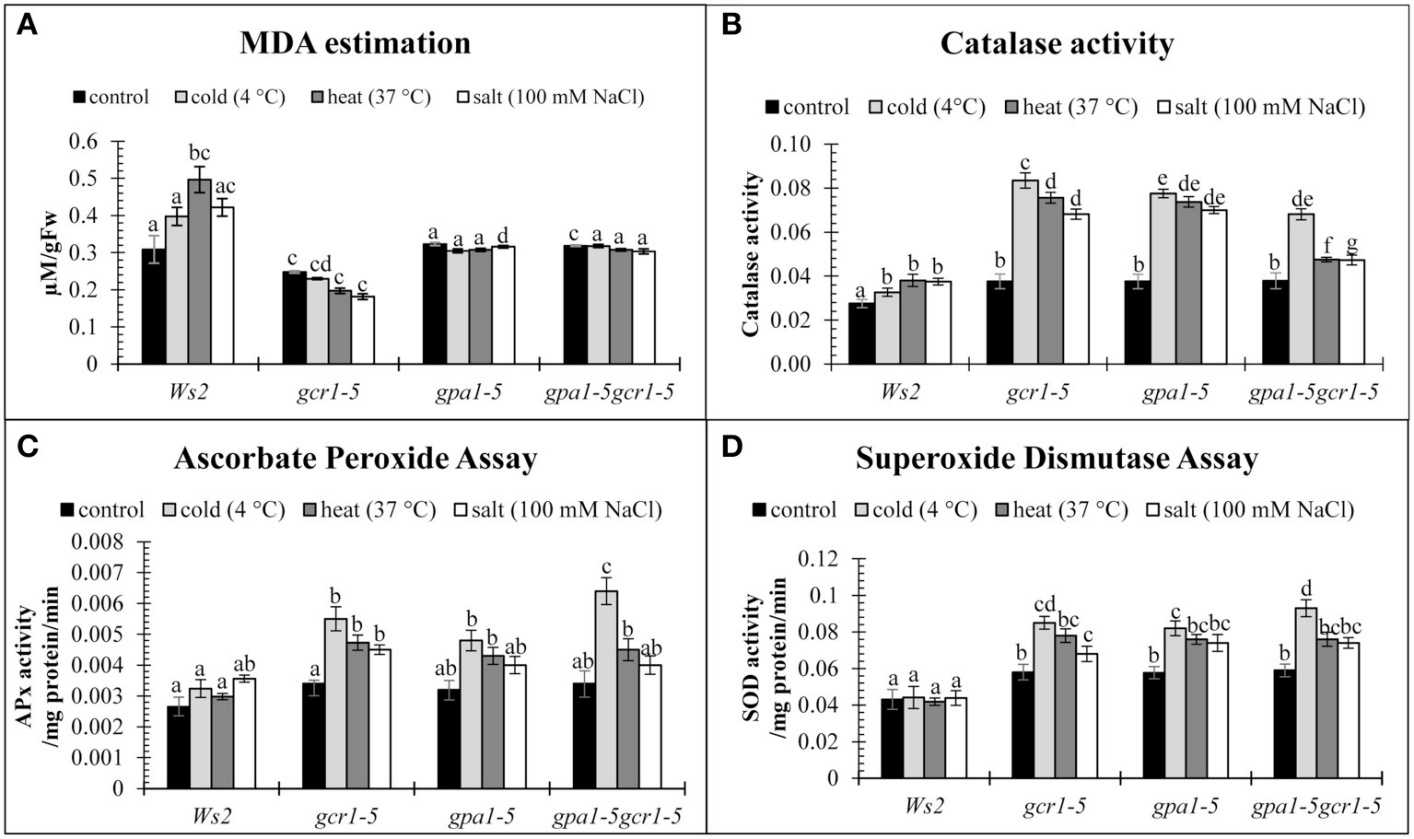
**Enzymatic stress markers, MDA (A), Catalase assay (B), Ascorbate peroxidase assay (C), and Superoxide dismutase assay (D) to validate the roles of GCR1/GPA1 in abiotic stress**. The values are mean of 3 independent experiments each having technical triplicates. Data is represented as mean ± SE. Values followed by different letters are significantly different at 5% level as determined by Duncan's test.

In this study, MDA was found to be much higher in the stressed WT plants than in any of the three mutants (Figure 2B), suggesting higher membrane injury and accumulation of free radicals in the WT plants. Even in the absence of any stress, *gcr1-5* mutant showed lower amount of MDA than the WT. However, the other mutants, *gpa1-5* and *gpa1-5gcr1-5* did not show significant difference in their levels of MDA relative to WT (Figure 5A). We also assayed other stress-related enzymes, catalase, ascorbate peroxidase, and superoxide dismutase, in both wild-type and the three mutants under control and stress treatments. In the absence of any stress, the activities of all these enzymes were similar in the WT and all the three mutants. When exposed to stress, these enzyme levels increased in the mutants under all the stresses tested, with maximum activities under cold stress followed by heat and salt, while there was no significant change in the wild type (Figures 5B–D).

## Discussion

The involvement G-protein α subunit in plants in individual abiotic stress responses is either known directly in relation to heat (Misra et al., 2007) and salt (Colaneri et al., 2014; Urano et al., 2014) or indirectly in relation to ABA signaling (Pandey et al., 2010; Alvarez et al., 2011) or oxidative stress (Booker et al., 2012). In addition, the β subunit has been implicated in heat response in pea (Misra et al., 2007), whereas γ subunit has only been implicated in biotic stress so far (Trusov et al., 2007; Trusov and Botella, 2012; Thung et al., 2013). However, comprehensive and/or comparative assessment of the involvement of any heterotrimeric G-protein subunit in all the major abiotic stresses has not been tested in any single plant so far. The best known candidate for a plant G-protein coupled receptor, the *Arabidopsis* GCR1 was implicated in drought stress (Pandey and Assmann, 2004), but the annotation of GCR1 as a GPCR, its interaction with GPA1 as well as its role in G-protein signaling was contested (Urano et al., 2013; Urano and Jones, 2013). This made it difficult to link any role of GCR1 in abiotic stress with that of G-protein signaling.

### Functional genomic identification and qPCR validation of the role of G-protein signaling components in abiotic stress

Our parallel transcriptome analyses of *Arabidopsis* single and double mutants of GCR1 and GPA1 (Chakraborty et al., 2015a,b; Chakraborty et al., submitted) under identical conditions gave the strongest indication of their substantial partnership on a genomewide basis for the first time, including in stress. Response to stress and response to stimulus emerged as the largest affected process in the transcriptomes of the single mutants of *gpa1-5, gcr1-5* as well as the double mutant *gpa1-5gcr1-5*. This not only revived the role of GCR1-GPA1 partnership in regulating a number of genes and an even higher number of processes, but also indicated that GCR1 and GPA1 may also work independently, possibly with other GCR/GPA isoforms or entirely different partners, to regulate some of the genes. The indication that genes related to stress-response figured in both shared and independent categories led us to hypothesize that G-protein signaling components may be the common conduits for responding to multiple stresses and could therefore be attractive targets for developing stress tolerance. In order to test this hypothesis, we thoroughly examined the stress-related DEGs *in silico*, and also validated them experimentally in a comprehensive manner in the present study. The experimental validation was done by investigating the impact of different stresses on the wild type, single and double mutants of GCR1 and GPA1 simultaneously under identical conditions for the first time.

Our comparison of DEGs from GPA1 and/or GCR1-responsive transcriptomes revealed 144 DEGs spanning a wide range of abiotic stresses, including heat, cold, salt, light stress etc. (Supplementary Table 1). Out of them, only 10 DEGs are shared by all the three mutants. Interestingly, each of the single mutants shared many more DEGs with the double mutant than between themselves (Figure 1). RT-qPCR validation of 28 of these genes spanning different stresses revealed identical regulation of the DEGs shared between the mutants (Figure 2). This can be best explained by GCR1-GPA1 partnership in regulating abiotic stress response in a classical G-protein signaling pathway. The seemingly independent regulation of the remaining un-shared DEGs between the 3 mutants could either be due to the GCR1/GPA1 partnership with other (known or unknown) GPA/GCR isoforms, or entirely different signaling pathways.

### GCR1 and/or GPA1 mutants are tolerant to multiple abiotic stresses

Plants have specialized regulatory networks which mediate sensing, response and adjustment of plant to change in environmental conditions such as change in temperature, amount of water, presence of salt and other minerals, etc. (Bailey-Serres et al., 2012). These networks are also linked to gene networks related to plant growth (Hirayama and Shinozaki, 2010). Therefore, we sought to validate the stress-related gene clusters predicted from our mutants by testing their physiological and biochemical response to stress. This was done by exposing the wild type, single and double mutants parallelly to three different stresses, *viz*. salt (100 mM NaCl), cold (4°C) and heat (37°C). Out of all the 3 stresses checked, heat and cold caused significant reduction in germination in all while the effect of cold was minimal (Figure 3A). The effect of all the stresses on root length in all wild type and the mutants was similar (Figure 3B). Effect on the shoot length under different stress was similar to that observed in % germination (Figure 3C), with heat and salt causing drastic reduction of shoot length. The only difference observed was that the single mutants had longer shoots than the double mutant under cold stress. All these results suggest that the mutants were able to withstand the stress conditions better than the wild type. This not only confirms our hypothesis that G-proteins signaling components could mediate the plant's response to multiple stresses, but also prove that their knock-out mutation renders the plants more tolerant to multiple abiotic stresses. In other words, G-protein signaling may enhance the sensitivity of the plant to abiotic stresses.

This was further confirmed by studying the non-enzymatic and enzymatic stress markers. For example, relative water content (RWC), which influences water relations of a plant (Slavík, 1974), decreased to a much lesser extent under different stress conditions in the mutants than in the wildtype, with the *gcr1-5* mutant being more tolerant to any stress than others (Figure 4A), Moreover, all the 3 mutants are more tolerant to cold stress than heat or salt stress. Proline accumulation is widely accepted as an indicator of abiotic stress and higher levels of proline accumulation are associated with abiotic stress tolerance (Ashraf and Foolad, 2007). In our study, proline content was much higher in all the three mutants (relative to WT) in all the stresses, with all 3 mutants showing maximum proline accumulation under cold stress (Figure 4B).

Lipid peroxidation has been established as a major mechanism of cellular injury in many biological systems of plant and animal origin and is measured in units of MDA (Hodges et al., 1999). MDA is used as an index to measure membrane injury in plants under any stress. In this study, MDA was found to be much higher in the stressed WT plants than in the mutants (Figure 5A), suggesting higher membrane injury and accumulation of free radicals in the WT plants. Even in the absence of any stress, *gcr1-5* mutant showed lower amount of MDA than the WT. However, the other mutants, *gpa1-5* and *gpa1-5gcr1-5* did not show significant difference in their levels of MDA relative to WT.

During stress, plant cells produce large quantities of reactive oxygen species (ROS), which cause damage to protein, lipids and DNA (Schützendübel and Polle, 2002). Under normal conditions, the level of ROS remains low due to the presence of active free radical scavenging enzymes like superoxide dismutase (SOD), catalase, and ascorbate peroxidase. We assayed these enzymes all the three mutants and found that relative to WT, all of them have higher activity of these enzymes under control conditions. When exposed to stress, these enzyme levels increased even further in the mutants as compared to the wild type, with maximum activities under cold stress followed by heat and salt (Figures 5B–D). These results indicate that functional GPA1 and GCR1 may subdue the ROS-scavenging ability and make the plant stress-sensitive and build up ROS. Their loss of function in the mutants makes them more tolerant to stress by enhanced activity of ROS-scavenging enzymes.

Significantly, our transcriptome data on the single and double mutants revealed none of the genes coding for ROS scavenging enzymes as differentially regulated. This indicates the role of GCR1-GPA1-regulation of these enzymes at the post-translational level.

## Conclusions and prospects

Our detailed analysis of the stress related category of DEGs identified from our *Arabidopsis* whole transcriptome microarray data on the GPA1 and GCR1 single and double mutants, as well as their comprehensive parallel validation in response to cold, heat and salt stresses clearly confirms the roles of GCR1 and GPA1 in abiotic stress response for the first time. This implies a role for classical g-protein signaling pathways involving GCR1 and GPA1 in stress sensitivity in the normal plants of Arabidopsis. The identical response of each of the 3 mutants to each of the 3 stresses is striking, despite the fact that they do not share majority of the genes belonging to stress response in their transcriptomes (Chakraborty et al., submitted). Indeed, this is an ample proof of our recent prediction that even if all the 3 mutants do not share majority of their DEGs, they may achieve the same regulatory outcomes, wherever their unshared DEGs belong to shared biological processes (Chakraborty et al., submitted). Another important contribution of this paper is to revive the role of GCR-GPA coupling in abiotic stress signaling in Arabidopsis. Most importantly, our findings also offer G-protein signaling pathway as a potential source of novel common targets for the development of tolerance/resistance to multiple abiotic stresses. At the same time, it would be of interest to examine the genomewide response of these mutants to individual or combined stresses, so as to estimate what proportion of a genomewide stress response can be attributed to G-protein signaling. Efforts are underway.

### Conflict of interest statement

The handling editor Girdhar K. Pandey declares that, despite being affiliated with the same institute as the author Kanwaljeet Kaur, the review process was handled objectively. The authors declare that the research was conducted in the absence of any commercial or financial relationships that could be construed as a potential conflict of interest.

## Supplementary material

Supplementary Figure S1 *****In-silico*** analysis of all abiotic stress responsive genes in ***gcr1-5*** using Expression Browser**. It shows the expression value for each genes in a color coded form obtained from all experiment categories, plant growth stages, tissues types, treatments, and identifiers, and thumbnail summary of expression levels and cluster results.

Supplementary Figure S2 *****In-silico*** analysis of all abiotic stress responsive genes in ***gpa1-5*** using Expression Browser**. It shows the expression value for each genes in a color coded form obtained from all experiment categories, plant growth stages, tissues types, treatments, and identifiers, and thumbnail summary of expression levels and cluster results.

Supplementary Figure S3 *****In-silico*** analysis of all abiotic stress responsive genes in ***gpa1-5gcr1-5*** using Expression Browser**. It shows the expression value for each genes in a color coded form obtained from all experiment categories, plant growth stages, tissues types, treatments, and identifiers, and thumbnail summary of expression levels and cluster results.

Supplementary Table S1 **List of genes used for validation of stress response data, with their primer sequences and efficiencies**.

Supplementary Table S2 **Distribution of GPA1/GCR1 responsive genes in different abiotic stresses**. It shows the distribution of DEGs in the transcriptome of the single and double mutants of GPA1 and GCR1 (*gpa1-5, gcr1-5*, and *gpa1-5gcr1-5*) in different abiotic stresses.

Supplementary Table S3 **qPCR of stress responsive genes validate the role GPA1 and GCR1 in regulating abiotic stresses**. These genes have been implicated in various abiotic stress response previously and also found to be differentially regulated in our transcriptome data (GEO accession no. GSE 40217). The values are given as average of log2 fold change ±SE obtained from 3 independent experiments each having technical triplicates. Values followed by different letters are significantly different at 5% level as determined by Duncan's test.

## Abbreviations

GPA1: G-protein α subunit 1
GCR1: G-protein Coupled Receptor 1
DEGs: Differentially Expressed Genes
MDA: Malondialdehyde
RWC: Relative Water Content
APx: Ascorbate Peroxidase
SOD: Superoxide Dismutase.
