# Supplementary figures and images for: G-protein Signaling Components GCR1 and GPA1 Mediate Responses to Multiple Abiotic Stresses in Arabidopsis
Source: Front Plant Sci. 2015 Nov 18;6:1000. doi: 10.3389/fpls.2015.01000 (PMC4649046; doi:10.3389/fpls.2015.01000)

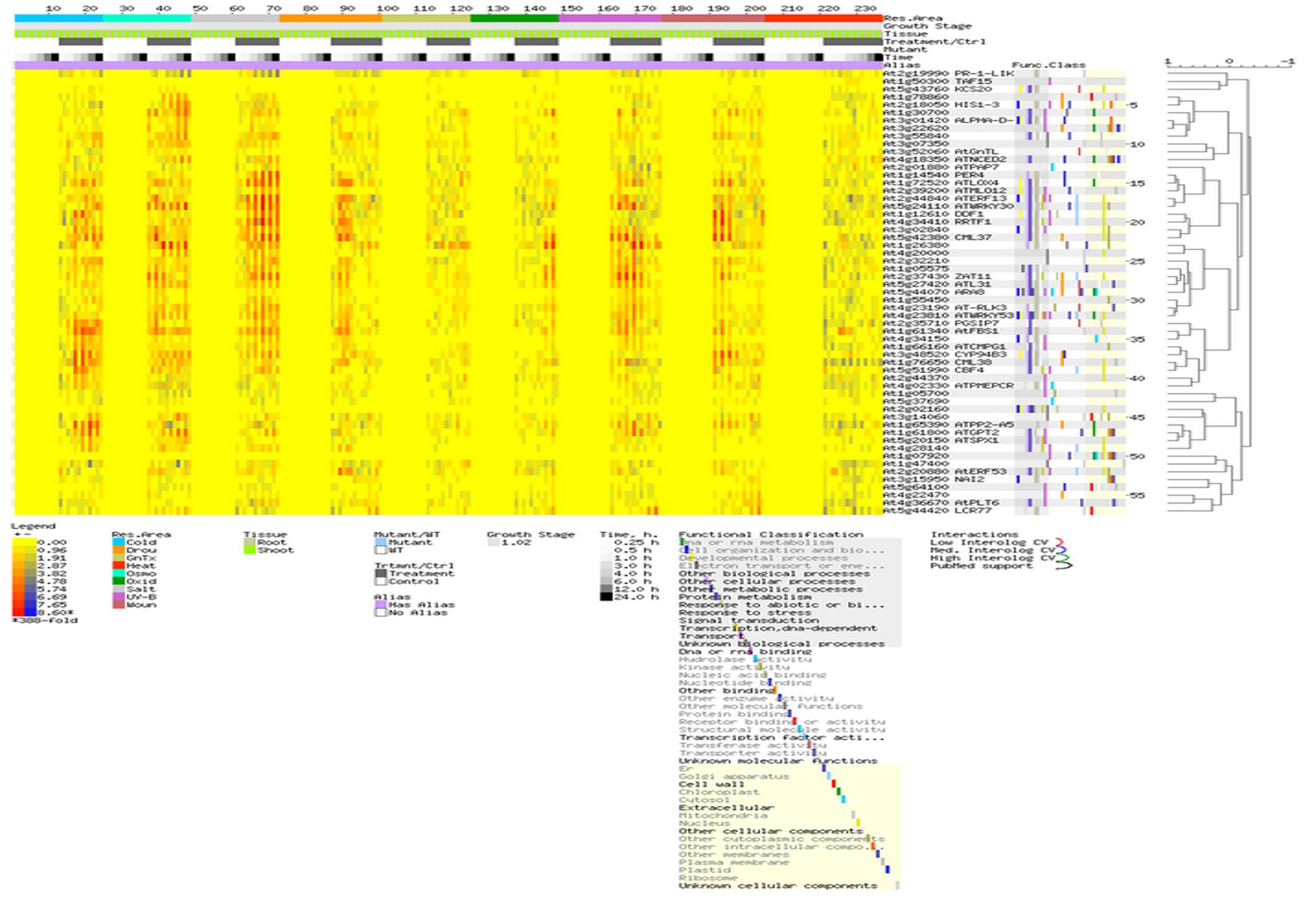

Supplement: Supplementary Figure S1 — In-silico analysis of all abiotic stress responsive genes in gcr1-5 using Expression Browser. It shows the expression value for each genes in a color coded form obtained from all experiment categories, plant growth stages, tissues types, treatments, and identifiers, and thumbnail summary of expression levels and cluster results. [file Image1.TIF]

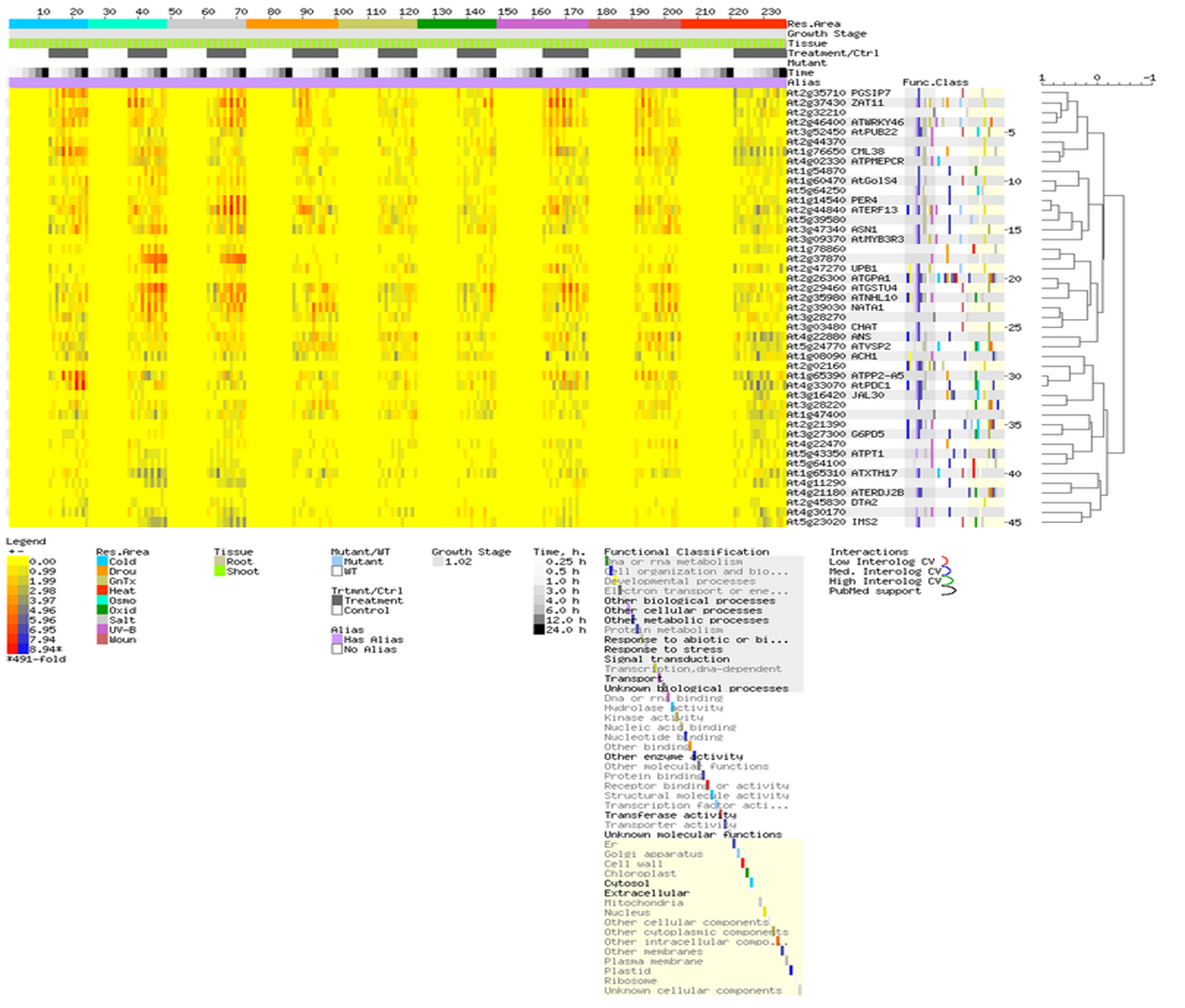

Supplement: Supplementary Figure S2 — In-silico analysis of all abiotic stress responsive genes in gpa1-5 using Expression Browser. It shows the expression value for each genes in a color coded form obtained from all experiment categories, plant growth stages, tissues types, treatments, and identifiers, and thumbnail summary of expression levels and cluster results. [file Image2.TIF]

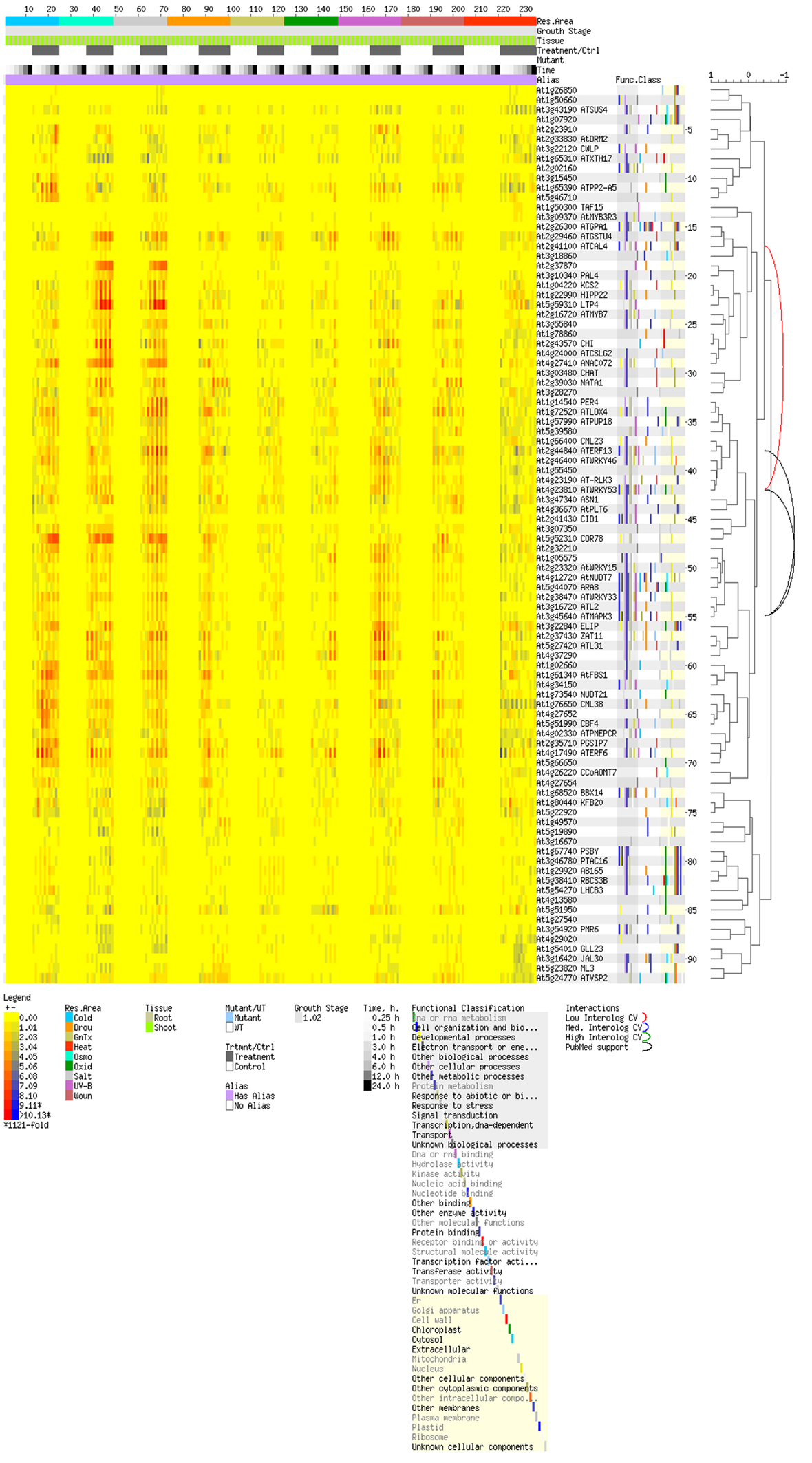

Supplement: Supplementary Figure S3 — In-silico analysis of all abiotic stress responsive genes in gpa1-5gcr1-5 using Expression Browser. It shows the expression value for each genes in a color coded form obtained from all experiment categories, plant growth stages, tissues types, treatments, and identifiers, and thumbnail summary of expression levels and cluster results. [file Image3.TIF]
