# Supplementary material for: G-protein Signaling Components GCR1 and GPA1 Mediate Responses to Multiple Abiotic Stresses in Arabidopsis
Source: Front Plant Sci. 2015 Nov 18;6:1000. doi: 10.3389/fpls.2015.01000 (PMC4649046; doi:10.3389/fpls.2015.01000)
Supplement: Supplementary Table S1 — List of genes used for validation of stress response data, with their primer sequences and efficiencies. [file Table1.DOCX]

**Supplementary Table S1. List of genes used for validation of stress response data, with their primer sequences and efficiencies.**

| Locus ID | Gene Name | Forward Primer | Reverse Primer | Efficiency (%) |
| --- | --- | --- | --- | --- |
| At1g66160 | CMPG1 | GCATGGAGATGGTTACGAAGA | GTCGAAGCAAACGCAAAGAA | 96.8 |
| At5g20150 | SPX1 | ATCTTCCCTGCTAACGAAAC | CAGCGATTGTGCTCTTCA | 97.8 |
| At2g35980 | YLS9 | CAAGTTCAGGCTTAGGGTTAGG | AGGTAGTTGTAGTTCCGTTTGAG | 104.4 |
| At5g24770 | VSP2 | GCGTACTGGTTGTGGTTAG | GTTCAATCCCGAGCTCTATG | 98.5 |
| At1g04220 | KCS2 | AATCCGACGCCTTCGTTATC | ACCAGCACTACAACCCATTC | 99.4 |
| At2g37430 | ZAT11 | CATGAGACGGCATAGGTCAAG | CTTACTACTACCGCATCGTTTCA | 98.9 |
| At2g46400 | WRKY46 | ACCAAGTCCGAAGAAGTGATG | CCACACACCGTTCTCAATCT | 100.2 |
| At2g39200 | MLO12 | GCATCTCCTCCACAACTACAA | CTTGGCCGCTATAATCAGAGAA | 96.4 |
| At5g19890 | Peroxidase superfamily protein | GACGTCGTAGCTTTATCAGGAG | AGTGTTGCGTCGGGATTT | 104.3 |
| At3g47340 | ASN1 | ATTGGTGTCACTTCGCTCTAC | TGCTCACAGTCATCGTTTAGG | 98.8 |
| At3g22840 | ELIP1 | ATTATCCGGTGGGAGTGAGA | TTCATAGGAGGAGGAGGAGATG | 90.8 |
| At4g27410 | RD26 | CTAGCTCCGACCAATGGATTAC | GAGTTCTGCTGCCGATTCA | 96.6 |
| At1g55450 | S-adenosyl-L-methionine-dependent methyltransferases superfamily protein | CCATCACACTCCCACAACAA | CTTGTGCTGCCACGATTAGA | 94.8 |
| At4g30170 | Peroxidase family protein | TCAGGAGCACACACTATAGGA | GAACCACGTATCCACGGTTTA | 97.3 |
| At5g44420 | PDF1.2 | AATGGTGGAAGCACAGAAG | TGGCTCCTTCAAGGTTAATG | 104.5 |
| At4g17490 | ERF6 | CGAGGATCAAAGGCGATTCT | CCGTCTCTCTTCCGTTTGTT | 93.8 |
| At1g72520 | LOX4 | GGAAATCGGACGGATAGAGAAG | CTCGGAGCCATCAACTCATAC | 99.4 |
| At1g08090 | NRT2 | CCTCTGTCTCTGGGAGTATCTT | GGCAGACAACATCACAAGGA | 102.0 |
| At4g34410 | RRTF1 | CCAGTGACAGCACAGTGATAAG | AGTTACAGCCGAGACATCCT | 99.9 |
| At4g22880 | LDOX | GGCCTAAGACACCAAGTGATTA | AAACCTAGACCGACAGAGAGA | 93.7 |
| At2g44840 | ERF13 | ACGATAACTGGAGCGACTTG | GCATCACGGAGAGTGTTGTA | 96.0 |
| At5g42380 | CML37 | CTACAAAGCTGCGTTAGTCTCT | CTATAAACCCGTCTCCGTCAAC | 106.4 |
| At5g27420 | CNI1 | TAAAGGAGCGTTGGAGTGTG | CGCCGATACAATGAGGATGAA | 91.9 |
| At5g59310 | LTP4 | GCTTTCGCTTTGAGGTTCTTC | CCACTGTGCCACATGTTATTG | 93.6 |
| At1g65390 | ATPP2-A5 | CGCTTCCTCTTCATGGACTATAC | ATGCGAGCGAGACACTAAAG | 100.3 |
| At1g76650 | CML38 | GCCGTAGCTGCTGTTAGATT | CCTTGAGCTCCATCTTCTTCTC | 99.8 |
| At5g52310 | RD29A | CACCAGGCGTAACAGGTAAA | GGAAGACACGACAGGAAACA | 99.7 |
| At4g23190 | CRK11 | ACGTAAATCGCCGTCTCATC | CGGTTCGGTTGTTGTCCTAT | 95.9 |

**Supplementary Table S2. Distribution of GPA1/GCR1 resposive genes in different abiotic stresses.** It shows the distribution of DEGs in the transcriptome of the single and double mutants of GPA1 and GCR1 (*gpa1-5*, *gcr1-5* and *gpa1-5gcr1-5*) in different abiotic stresses.

| Abiotic Stress | *gpa1-5* | *gcr1-5* | *gpa1-5gcr1-5* |
| --- | --- | --- | --- |
| Cold | At4g02330 | At2g18050 | At4g02330 |
|  | At1g65390 | At4g02330 | At4g27410 |
|  | At5g39580 | At1g05700 | At5g46710 |
|  | At1g78860 | At1g65390 | At2g43570 |
|  | At3g47340 | At1g78860 | At1g65390 |
|  | At1g65310 | At4g23190 | At1g80440 |
|  | At1g60470 | At1g76650 | At5g39580 |
|  | At4g11290 | At2g35710 | At1g78860 |
|  | At1g76650 | At2g39200 | At3g22840 |
|  | At2g35710 | At1g61340 | At1g54010 |
|  | At4g33070 | At2g44840 | At3g47340 |
|  | At1g08090 | At2g02160 | At1g65310 |
|  | At2g44840 | At1g12610 | At5g54270 |
|  | At2g02160 | At4g23810 | At1g76650 |
|  | At5g64100 | At5g64100 | At2g35710 |
|  | At4g30170 | At5g51990 | At1g61340 |
|  | At3g27300 | At1g66160 | At5g23820 |
|  | At4g22470 | At1g30700 | At2g02160 |
|  | At2g32210 | At4g36670 | At4g23810 |
|  | At3g09370 | At2g32210 | At5g52310 |
|  |  | At4g22470 | At4g36670 |
|  |  |  | At2g32210 |
|  |  |  | At5g19890 |
|  |  |  | At1g49570 |
|  |  |  | At3g22120 |
|  |  |  | At1g22990 |
|  |  |  | At2g41100 |
|  |  |  | At4g23190 |
|  |  |  | At4g29020 |
|  |  |  | At4g13580 |
|  |  |  | At2g44840 |
|  |  |  | At4g17490 |
|  |  |  | At5g66650 |
|  |  |  | At5g51990 |
|  |  |  | At2g38470 |
|  |  |  | At5g38410 |
|  |  |  | At4g26220 |
|  |  |  | At3g46780 |
|  |  |  | At3g48340 |
|  |  |  | At3g09370 |
|  |  |  | At1g04220 |
| Heat | At4g21180 | At5g27420 | At5g27420 |
|  | At1g76650 | At1g76650 | At1g76650 |
|  |  |  | At4g37290 |
|  |  |  | At2g23910 |
|  |  |  | At4g24000 |
| Salt | At4g02330 | At2g18050 | At4g02330 |
|  | At1g65390 | At4g02330 | At4g27410 |
|  | At5g39580 | At3g48520 | At5g44070 |
|  | At1g78860 | At1g05700 | At1g65390 |
|  | At2g37870 | At5g44070 | At5g39580 |
|  | At3g28220 | At4g28140 | At1g78860 |
|  | At3g47340 | At3g52060 | At1g54010 |
|  | At4g11290 | At1g65390 | At3g48390 |
|  | At2g35710 | At2g20880 | At1g67740 |
|  | At5g24770 | At1g78860 | At3g47340 |
|  | At5g64250 | At4g23190 | At5g54270 |
|  | At2g26300 | At2g35710 | At2g35710 |
|  | At1g08090 | At3g07350 | At5g24770 |
|  | At4g22470 | At1g26380 | At2g41430 |
|  | At2g29460 | At4g22470 | At3g43190 |
|  |  |  | At5g52310 |
|  |  |  | At5g51950 |
|  |  |  | At5g19890 |
|  |  |  | At1g49570 |
|  |  |  | At3g54920 |
|  |  |  | At2g37870 |
|  |  |  | At2g41100 |
|  |  |  | At3g15450 |
|  |  |  | At4g23190 |
|  |  |  | At1g02660 |
|  |  |  | At2g16720 |
|  |  |  | At2g23320 |
|  |  |  | At2g26300 |
|  |  |  | At1g29920 |
|  |  |  | At4g13580 |
|  |  |  | At3g07350 |
|  |  |  | At3g18860 |
|  |  |  | At5g38410 |
|  |  |  | At4g24000 |
|  |  |  | At2g29460 |
|  |  |  | At1g04220 |
| Drought | At3g03480 | At1g72520 | At1g72520 |
|  | At2g39030 | At3g48520 | At4g27410 |
|  | At1g54870 | At5g42380 | At1g68520 |
|  | At2g37870 | At2g19990 | At2g39030 |
|  | At3g28220 | At4g28140 | At5g46710 |
|  | At3g47340 | At2g20880 | At2g43570 |
|  | At1g76650 | At3g01420 | At2g33830 |
|  | At2g44840 | At1g61800 | At5g59310 |
|  | At3g16420 | At1g76650 | At1g80440 |
|  | At3g28270 | At5g44420 | At3g48390 |
|  | At2g29460 | At1g61340 | At1g67740 |
|  |  | At2g44840 | At3g47340 |
|  |  | At5g43760 | At1g76650 |
|  |  | At1g12610 | At1g61340 |
|  |  | At5g51990 | At2g41430 |
|  |  | At3g22620 | At3g43190 |
|  |  | At1g55450 | At5g52310 |
|  |  |  | At3g28270 |
|  |  |  | At1g55450 |
|  |  |  | At3g03480 |
|  |  |  | At3g45640 |
|  |  |  | At3g22120 |
|  |  |  | At2g37870 |
|  |  |  | At1g02660 |
|  |  |  | At2g23320 |
|  |  |  | At2g44840 |
|  |  |  | At1g27540 |
|  |  |  | At5g66650 |
|  |  |  | At5g51990 |
|  |  |  | At3g16420 |
|  |  |  | At2g38470 |
|  |  |  | At5g38410 |
|  |  |  | At3g16720 |
|  |  |  | At2g29460 |
| ABA | At4g21180 | At3g48520 | At4g27410 |
|  | At3g47340 | At5g20150 | At4g12720 |
|  | At5g24770 | At4g18350 | At5g59310 |
|  | At2g26300 | At3g14060 | At3g47340 |
|  | At2g02160 | At2g02160 | At5g22920 |
|  | At2g45830 | At1g66160 | At5g24770 |
|  | At3g16420 | At3g15950 | At5g23820 |
|  |  |  | At2g41430 |
|  |  |  | At2g02160 |
|  |  |  | At3g43190 |
|  |  |  | At5g52310 |
|  |  |  | At2g23910 |
|  |  |  | At1g02660 |
|  |  |  | At2g23320 |
|  |  |  | At2g26300 |
|  |  |  | At1g29920 |
|  |  |  | At3g16420 |
|  |  |  | At4g24000 |
| Light | At2g35980 | At3g02840 | At4g27652 |
|  | At3g52450 | At3g55840 | At4g27410 |
|  | At1g60470 | At5g44070 | At4g27654 |
|  | At1g14540 | At5g27420 | At5g44070 |
|  | At5g24770 | At4g23190 | At5g27420 |
|  | At2g37430 | At1g14540 | At3g16670 |
|  | At2g37430 | At1g61340 | At1g14540 |
|  | At4g22880 | At1g12610 | At5g24770 |
|  |  | At4g34410 | At1g61340 |
|  |  | At3g07350 | At1g73540 |
|  |  | At2g37430 | At5g52310 |
|  |  | At1g07920 | At1g07920 |
|  |  | At4g20000 | At1g50300 |
|  |  | At1g50300 | At3g55840 |
|  |  | At5g24110 | At1g66400 |
|  |  |  | At4g23190 |
|  |  |  | At1g02660 |
|  |  |  | At2g23320 |
|  |  |  | At3g07350 |
|  |  |  | At3g16420 |
|  |  |  | At2g37430 |
|  |  |  | At1g50660 |
| UV-B | At5g43350 | At3g02840 | At5g46710 |
|  | At2g47270 | At5g27420 | At5g27420 |
|  | At1g76650 | At1g76650 | At1g76650 |
|  | At2g46400 | At1g05575 | At4g37290 |
|  | At2g37430 | At1g61340 | At1g05575 |
|  | At2g44370 | At2g37430 | At1g61340 |
|  |  | At2g44370 | At2g46400 |
|  |  | At5g24110 | At5g66650 |
|  |  |  | At2g37430 |
|  |  |  | At3g16720 |
| Metal | At2g35980 | At4g34150 | At1g80440 |
|  | At2g21390 | At5g37690 | At4g34150 |
|  | At1g47400 | At1g47400 | At3g10340 |
|  | At5g23020 | At2g01880 | At1g57990 |
|  |  |  | At1g26850 |

**Supplementary table S3:** **qPCR of stress responsive genes validate the role GPA1 and GCR1 in regulating abiotic stresses.** These genes have been implicated in various abiotic stress response previously and also found to be differentially regulated in our transcriptome data (GEO accession no. GSE 40217). The values are given as average of log2 fold change±SE obtained from 3 independent experiments each having technical triplicates. Values followed by different letters are significantly different at 5 % level as determined by Duncan’s test.

|  | Control | | | | | cold (4 °C) | | | | heat (37 °C) | | | | NaCl (100 mM) | | | |
| --- | --- | --- | --- | --- | --- | --- | --- | --- | --- | --- | --- | --- | --- | --- | --- | --- | --- |
|  | **Ws2** | ***gcr1-5*** | ***gpa1-5*** | ***gpa1-5gcr1-5*** | **Ws2** | | ***gcr1-5*** | ***gpa1-5*** | ***gpa1-5gcr1-5*** | **Ws2** | ***gcr1-5*** | ***gpa1-5*** | ***gpa1-5gcr1-5*** | **Ws2** | ***gcr1-5*** | ***gpa1-5*** | ***gpa1-5gcr1-5*** |
| ERF13 | 0.00±0.00a | 1.60±0.15b | 1.45±0.06c | 1.91±0.12b | 4.23±0.52a | | 5.13±0.32a | 5.93±0.20c | 6.10±0.12bc | -4.87±0.18ab | -2.20±0.06d | -3.24±0.09a | -1.88±0.12e | 9.53±0.35d | 11.90±0.17de | 11.37±0.21bd | 8.93±0.42ab |
| CML37 | 0.00±0.00a | 1.26±0.04b | 1.03±0.10a | 0.78±0.05b | 5.17±0.20bc | | 6.29±0.32b | 5.33±0.07c | 5.08±0.27bc | 2.23±0.09d | 4.00±0.28e | 3.10±0.21b | 3.65±0.30bd | 7.83±0.20de | 10.81±0.26d | 9.23±0.32e | 8.11±0.11f |
| RRTF1 | 0.00±0.00a | 4.00±0.12b | 0.69±0.04c | 0.51±0.02d | -3.60±0.31e | | 0.09±0.04be | -4.00±0.25d | -2.90±0.15b | -2.98±0.19bc | -1.97±0.28bd | -2.87±0.12f | -3.27±0.09de | 8.25±0.32f | 12.33±0.47f | 9.07±0.37d | 6.93±0.35c |
| LTP4 | 0.00±0.00a | -0.28±0.03b | -1.00±0.12c | -1.49±0.19c | -2.60±0.29d | | -2.67±0.29d | -3.16±0.29c | -4.29±0.08bc | 4.73±0.24bc | 4.23±0.32d | 2.94±0.24d | 2.10±0.12cd | 10.64±0.48e | 10.71±0.26e | 8.61±0.09e | 8.26±0.31e |
| WRKY46 | 0.00±0.00a | 1.06±0.09b | 1.31±0.07c | 1.09±0.06b | 3.87±0.22d | | 4.97±0.28e | 4.10±0.10e | 4.50±0.06d | -1.77±0.18bc | -1.73±0.23e | -2.47±0.17f | -3.03±0.22ef | 5.67±0.23e | 6.07±0.19de | 6.90±0.15de | 5.03±0.22g |
| RD26 | 0.00±0.00a | 0.74±0.05a | 0.43±0.04a | 1.15±0.08b | 6.47±0.28c | | 7.03±0.43d | 7.37±0.56c | 8.07±0.09cd | 2.70±0.25b | 3.97±0.28e | 3.97±0.28e | 3.13±0.18de | 5.10±0.26c | 4.90±0.25e | 6.07±0.23c | 5.93±0.27bc |
| ERF6 | 0.00±0.00a | 0.68±0.03a | 0.70±0.04a | 1.12±0.04b | 8.80±0.21c | | 9.07±0.24d | 8.10±0.17c | 11.47±0.35e | -6.73±0.24cd | -6.95±0.21cd | -5.57±0.27bc | -5.90±0.35bc | 5.40±0.21bc | 5.87±0.28bc | 5.97±0.28f | 6.00±0.15g |
| KCS2 | 0.00±0.00a | -0.31±0.02a | -0.44±0.07a | -1.10±0.07b | -3.43±0.19c | | -4.13±0.18bc | -2.41±0.21b | -4.27±0.28bc | 1.57±0.12b | 2.53±0.18d | 1.47±0.18b | 2.20±0.06b | 5.80±0.23bc | 6.07±0.19bc | 5.90±0.25b | 4.40±0.12c |
| CRK11 | 0.00±0.00a | 1.37±0.13b | 0.70±0.08a | 2.01±0.21c | -1.41±0.13d | | 0.15±0.01a | -2.18±0.06e | -2.16±0.43e | -1.58±0.26e | 0.39±0.02a | -2.73±0.19e | -1.85±0.17de | 5.14±0.21f | 9.81±0.10g | 5.74±0.28f | 7.27±0.30fg |
| YLS9 | 0.00±0.00a | -0.57±0.03a | -1.20±0.07b | -0.89±0.05a | -2.97±0.35c | | -3.87±0.18c | -4.53±0.18d | -4.57±0.15d | -2.10±0.17bc | -3.07±0.15c | -3.17±0.09bc | -4.10±0.32d | 5.07±0.39e | 4.83±0.20f | 5.30±0.29e | 3.85±0.19ef |
| RD29A | 0.00±0.00a | 0.44±0.03a | 0.48±0.03a | 1.11±0.06b | 7.67±0.29c | | 7.49±0.32c | 7.28±0.45bc | 8.61±0.27bc | 2.23±0.16b | 2.78±0.23b | 2.84±0.20bc | 5.54±0.30c | 5.89±0.18d | 5.14±0.31cd | 5.15±0.20d | 6.87±0.31e |
| AT-PP2A5 | 0.00±0.00a | 1.92±0.11a | 1.13±0.06b | 2.04±0.16a | 7.73±0.33c | | 9.51±0.29bc | 8.90±0.34d | 10.07±0.11bd | -3.80±0.23e | -1.99±0.16f | -1.31±0.09a | -1.79±0.28ef | 2.61±0.32a | 4.19±0.24ab | 5.06±0.17c | 5.97±0.38cd |
| LOX4 | 0.00±0.00a | 1.33±0.06b | 0.58±0.03a | 1.57±0.09b | 5.10±0.12c | | 7.03±0.12bc | 5.47±0.18c | 5.97±0.28c | -2.20±0.15d | -2.90±0.25d | -2.63±0.28bd | -2.97±0.19 | 6.90±0.26bc | 7.93±0.24c | 5.03±0.22b | 9.83±0.14bd |
| ELIP1 | 0.00±0.00a | 0.09±0.04a | -0.44±0.06a | -1.03±0.04b | 4.37±0.15c | | 4.20±0.06c | 2.10±0.12d | 2.87±0.22d | 3.63±0.30cd | 3.63±0.30d | 2.97±0.28c | 2.73±0.29cd | 4.73±0.62c | 4.93±0.17c | 5.00±0.42cd | 4.40±0.12d |
| CML38 | 0.00±0.00a | 1.29±0.04b | 1.35±0.06b | 1.65±0.07c | 7.53±0.48d | | 7.89±0.39d | 7.92±0.17c | 9.26±0.34bd | -4.36±0.42e | -4.23±0.32f | -2.68±0.28ef | -2.04±0.41e | 1.89±0.21b | 3.67±0.29d | 4.17±0.23bd | 1.65±0.07bc |
| LDOX | 0.00±0.00a | -0.43±0.06a | -2.65±0.24b | -1.95±0.08b | -2.63±0.38b | | -3.53±0.18c | -5.03±0.19d | -4.19±0.22bc | -4.70±0.26bc | -5.43±0.19d | -5.94±0.24b | -4.09±0.12bd | 3.07±0.20e | 1.89±0.14f | 4.03±0.33g | 4.11±0.25g |
| CNI1 | 0.00±0.00a | 1.26±0.11b | 0.71±0.08a | 1.25±0.12b | 1.90±0.21b | | 2.45±0.10c | 2.92±0.24c | 4.15±0.26bc | 2.06±0.18c | 3.87±0.38bc | 1.77±0.26d | 5.97±0.31cd | 3.30±0.15cd | 4.09±0.33d | 3.79±0.24d | 5.29±0.22cd |
| At1g55450 | 0.00±0.00 | 1.24± 0.06b | 0.65± 0.03a | 1.29± 0.01b | 3.03± 0.27c | | 3.93± 0.37c | 3.13± 0.03d | 5.07± 0.19bc | -2.50± 0.40e | -4.87± 0.34f | -2.93± 0.23e | -2.83± 0.19 | 3.67± 0.29bd | 5.07± 0.19bd | 3.70± 0.26d | 6.10± 0.25c |
| ZAT11 | 0.00±0.00a | 2.22± 0.20b | 1.33± 0.04c | 2.31± 0.06b | 2.40± 0.32b | | 5.20± 0.26bc | 3.93± 0.27d | 5.07± 0.19bd | -3.10± 0.20e | -1.60± 0.35f | -3.90± 0.25d | -2.07± 0.19g | 6.43± 0.46d | 8.23± 0.26bd | 3.60± 0.12b | 6.87± 0.18bc |
| CMPG1 | 0.00±0.00a | 1.39± 0.11b | 0.82± 0.06a | 0.70± 0.03a | 3.84± 0.26c | | 5.09± 0.11d | 5.10± 0.06d | 4.13± 0.03c | -1.52± 0.14e | -2.27± 0.09f | -2.17± 0.03f | -2.73± 0.20ef | 2.93± 0.24c | 5.79± 0.22cd | 3.07± 0.14d | 3.07± 0.24d |
| MLO12 | 0.00±0.00a | 2.21± 0.06b | 0.89± 0.05a | 1.10± 0.06b | 1.50± 0.05c | | 4.37± 0.12d | 1.83± 0.15b | 2.35± 0.04b | 1.15± 0.05c | 5.53± 0.28cd | 1.43± 0.12bc | 1.63± 0.12bc | 3.75± 0.16d | 6.97± 0.28e | 2.90± 0.25b | 3.17± 0.28cd |
| VSP2 | 0.00±0.00a | -0.02± 0.01a | -1.19± 0.21b | -1.17± 0.05b | -2.03± 0.15c | | -2.10± 0.21c | -3.03± 0.23d | -3.03± 0.32d | -2.37± 0.29 | -2.40± 0.38c | -3.33± 0.22cd | -3.03± 0.18bd | 2.17± 0.19e | 2.10± 0.21e | 1.73± 0.20f | 1.97± 0.19e |
| NRT2 | 0.00±0.00a | 1.07± 0.08b | 2.00± 0.07b | 0.16± 0.01a | -2.87± 0.24c | | -3.89± 0.17d | -2.14± 0.09c | -2.97± 0.34c | -5.13± 0.58e | -5.06± 0.43e | -2.99± 0.17cd | -4.74± 0.29d | -3.00± 0.38cd | -4.02± 0.20bd | 0.15± 0.02a | -2.88± 0.29e |
| SPX1 | 0.00±0.00a | -1.19± 0.10b | -0.54± 0.03a | -0.42± 0.04a | 3.23± 0.20c | | 2.03± 0.23d | 2.77± 0.23d | 2.90± 0.21cd | -2.10± 0.21ab | -2.77± 0.23b | -2.70± 0.21bc | -2.83± 0.15b | -1.10± 0.15e | -2.37± 0.18be | -1.60± 0.12e | -1.70± 0.15be |
| PDF1.2 | 0.00±0.00a | 1.53± 0.05b | 0.37± 0.03a | -0.56± 0.03c | -1.73± 0.28d | | -3.93± 0.32e | -1.87± 0.12d | -2.07± 0.15bd | -3.87± 0.29de | -5.80± 0.23f | -4.13± 0.20ef | -4.07± 0.19f | -2.13± 0.26d | -3.67± 0.29f | -2.09± 0.12d | -2.37± 0.15d |
| ASN1 | 0.00±0.00a | -0.72± 0.05a | 1.24± 0.05b | 1.79± 0.03b | -3.67± 0.29c | | -4.20± 0.06d | -2.09± 0.10e | -4.17± 0.26d | 2.47± 0.18b | 3.00± 0.20c | 2.27± 0.19bc | 3.90± 0.25e | -2.43± 0.27c | -2.63± 0.23cd | -3.37± 0.22d | -3.07± 0.20f |
| At4g30170 | 0.00±0.00a | 0.14± 0.03a | 1.59± 0.03b | 0.88± 0.05c | -2.73± 0.20d | | -2.70± 0.21d | -2.80± 0.35d | -2.77± 0.18d | -3.20± 0.25e | -3.17± 0.38de | -4.33± 0.33e | -3.07± 0.26d | -2.83± 0.23c | -3.13± 0.26b | -3.87± 0.23de | -3.03± 0.18bd |
| At5g19890 | 0.00±0.00a | 0.83± 0.05a | 0.77± 0.06a | 1.19± 0.10b | -1.50± 0.06c | | -2.23± 0.09d | -2.00± 0.15d | -2.33± 0.09c | -1.53± 0.30c | -2.37± 0.12cd | -1.50± 0.15d | -2.53± 0.13d | -4.50± 0.36e | -4.53± 0.18f | -4.97± 0.28g | -4.97± 0.19g |
