# Supplementary material for: G-protein Signaling Components GCR1 and GPA1 Mediate Responses to Multiple Abiotic Stresses in Arabidopsis
Source: Front Plant Sci. 2015 Nov 18;6:1000. doi: 10.3389/fpls.2015.01000 (PMC4649046; doi:10.3389/fpls.2015.01000)
Supplement: Supplementary Table S2 — Distribution of GPA1/GCR1 responsive genes in different abiotic stresses. It shows the distribution of DEGs in the transcriptome of the single and double mutants of GPA1 and GCR1 (gpa1-5, gcr1-5, and gpa1-5gcr1-5) in different abiotic stresses. [file Table2.DOCX]

**Supplementary Table S2. Distribution of GPA1/GCR1 resposive genes in different abiotic stresses.** It shows the distribution of DEGs in the transcriptome of the single and double mutants of GPA1 and GCR1 (*gpa1-5*, *gcr1-5* and *gpa1-5gcr1-5*) in different abiotic stresses.

| Abiotic Stress | *gpa1-5* | *gcr1-5* | *gpa1-5gcr1-5* |
| --- | --- | --- | --- |
| Cold | At4g02330 | At2g18050 | At4g02330 |
|  | At1g65390 | At4g02330 | At4g27410 |
|  | At5g39580 | At1g05700 | At5g46710 |
|  | At1g78860 | At1g65390 | At2g43570 |
|  | At3g47340 | At1g78860 | At1g65390 |
|  | At1g65310 | At4g23190 | At1g80440 |
|  | At1g60470 | At1g76650 | At5g39580 |
|  | At4g11290 | At2g35710 | At1g78860 |
|  | At1g76650 | At2g39200 | At3g22840 |
|  | At2g35710 | At1g61340 | At1g54010 |
|  | At4g33070 | At2g44840 | At3g47340 |
|  | At1g08090 | At2g02160 | At1g65310 |
|  | At2g44840 | At1g12610 | At5g54270 |
|  | At2g02160 | At4g23810 | At1g76650 |
|  | At5g64100 | At5g64100 | At2g35710 |
|  | At4g30170 | At5g51990 | At1g61340 |
|  | At3g27300 | At1g66160 | At5g23820 |
|  | At4g22470 | At1g30700 | At2g02160 |
|  | At2g32210 | At4g36670 | At4g23810 |
|  | At3g09370 | At2g32210 | At5g52310 |
|  |  | At4g22470 | At4g36670 |
|  |  |  | At2g32210 |
|  |  |  | At5g19890 |
|  |  |  | At1g49570 |
|  |  |  | At3g22120 |
|  |  |  | At1g22990 |
|  |  |  | At2g41100 |
|  |  |  | At4g23190 |
|  |  |  | At4g29020 |
|  |  |  | At4g13580 |
|  |  |  | At2g44840 |
|  |  |  | At4g17490 |
|  |  |  | At5g66650 |
|  |  |  | At5g51990 |
|  |  |  | At2g38470 |
|  |  |  | At5g38410 |
|  |  |  | At4g26220 |
|  |  |  | At3g46780 |
|  |  |  | At3g48340 |
|  |  |  | At3g09370 |
|  |  |  | At1g04220 |
| Heat | At4g21180 | At5g27420 | At5g27420 |
|  | At1g76650 | At1g76650 | At1g76650 |
|  |  |  | At4g37290 |
|  |  |  | At2g23910 |
|  |  |  | At4g24000 |
| Salt | At4g02330 | At2g18050 | At4g02330 |
|  | At1g65390 | At4g02330 | At4g27410 |
|  | At5g39580 | At3g48520 | At5g44070 |
|  | At1g78860 | At1g05700 | At1g65390 |
|  | At2g37870 | At5g44070 | At5g39580 |
|  | At3g28220 | At4g28140 | At1g78860 |
|  | At3g47340 | At3g52060 | At1g54010 |
|  | At4g11290 | At1g65390 | At3g48390 |
|  | At2g35710 | At2g20880 | At1g67740 |
|  | At5g24770 | At1g78860 | At3g47340 |
|  | At5g64250 | At4g23190 | At5g54270 |
|  | At2g26300 | At2g35710 | At2g35710 |
|  | At1g08090 | At3g07350 | At5g24770 |
|  | At4g22470 | At1g26380 | At2g41430 |
|  | At2g29460 | At4g22470 | At3g43190 |
|  |  |  | At5g52310 |
|  |  |  | At5g51950 |
|  |  |  | At5g19890 |
|  |  |  | At1g49570 |
|  |  |  | At3g54920 |
|  |  |  | At2g37870 |
|  |  |  | At2g41100 |
|  |  |  | At3g15450 |
|  |  |  | At4g23190 |
|  |  |  | At1g02660 |
|  |  |  | At2g16720 |
|  |  |  | At2g23320 |
|  |  |  | At2g26300 |
|  |  |  | At1g29920 |
|  |  |  | At4g13580 |
|  |  |  | At3g07350 |
|  |  |  | At3g18860 |
|  |  |  | At5g38410 |
|  |  |  | At4g24000 |
|  |  |  | At2g29460 |
|  |  |  | At1g04220 |
| Drought | At3g03480 | At1g72520 | At1g72520 |
|  | At2g39030 | At3g48520 | At4g27410 |
|  | At1g54870 | At5g42380 | At1g68520 |
|  | At2g37870 | At2g19990 | At2g39030 |
|  | At3g28220 | At4g28140 | At5g46710 |
|  | At3g47340 | At2g20880 | At2g43570 |
|  | At1g76650 | At3g01420 | At2g33830 |
|  | At2g44840 | At1g61800 | At5g59310 |
|  | At3g16420 | At1g76650 | At1g80440 |
|  | At3g28270 | At5g44420 | At3g48390 |
|  | At2g29460 | At1g61340 | At1g67740 |
|  |  | At2g44840 | At3g47340 |
|  |  | At5g43760 | At1g76650 |
|  |  | At1g12610 | At1g61340 |
|  |  | At5g51990 | At2g41430 |
|  |  | At3g22620 | At3g43190 |
|  |  | At1g55450 | At5g52310 |
|  |  |  | At3g28270 |
|  |  |  | At1g55450 |
|  |  |  | At3g03480 |
|  |  |  | At3g45640 |
|  |  |  | At3g22120 |
|  |  |  | At2g37870 |
|  |  |  | At1g02660 |
|  |  |  | At2g23320 |
|  |  |  | At2g44840 |
|  |  |  | At1g27540 |
|  |  |  | At5g66650 |
|  |  |  | At5g51990 |
|  |  |  | At3g16420 |
|  |  |  | At2g38470 |
|  |  |  | At5g38410 |
|  |  |  | At3g16720 |
|  |  |  | At2g29460 |
| ABA | At4g21180 | At3g48520 | At4g27410 |
|  | At3g47340 | At5g20150 | At4g12720 |
|  | At5g24770 | At4g18350 | At5g59310 |
|  | At2g26300 | At3g14060 | At3g47340 |
|  | At2g02160 | At2g02160 | At5g22920 |
|  | At2g45830 | At1g66160 | At5g24770 |
|  | At3g16420 | At3g15950 | At5g23820 |
|  |  |  | At2g41430 |
|  |  |  | At2g02160 |
|  |  |  | At3g43190 |
|  |  |  | At5g52310 |
|  |  |  | At2g23910 |
|  |  |  | At1g02660 |
|  |  |  | At2g23320 |
|  |  |  | At2g26300 |
|  |  |  | At1g29920 |
|  |  |  | At3g16420 |
|  |  |  | At4g24000 |
| Light | At2g35980 | At3g02840 | At4g27652 |
|  | At3g52450 | At3g55840 | At4g27410 |
|  | At1g60470 | At5g44070 | At4g27654 |
|  | At1g14540 | At5g27420 | At5g44070 |
|  | At5g24770 | At4g23190 | At5g27420 |
|  | At2g37430 | At1g14540 | At3g16670 |
|  | At2g37430 | At1g61340 | At1g14540 |
|  | At4g22880 | At1g12610 | At5g24770 |
|  |  | At4g34410 | At1g61340 |
|  |  | At3g07350 | At1g73540 |
|  |  | At2g37430 | At5g52310 |
|  |  | At1g07920 | At1g07920 |
|  |  | At4g20000 | At1g50300 |
|  |  | At1g50300 | At3g55840 |
|  |  | At5g24110 | At1g66400 |
|  |  |  | At4g23190 |
|  |  |  | At1g02660 |
|  |  |  | At2g23320 |
|  |  |  | At3g07350 |
|  |  |  | At3g16420 |
|  |  |  | At2g37430 |
|  |  |  | At1g50660 |
| UV-B | At5g43350 | At3g02840 | At5g46710 |
|  | At2g47270 | At5g27420 | At5g27420 |
|  | At1g76650 | At1g76650 | At1g76650 |
|  | At2g46400 | At1g05575 | At4g37290 |
|  | At2g37430 | At1g61340 | At1g05575 |
|  | At2g44370 | At2g37430 | At1g61340 |
|  |  | At2g44370 | At2g46400 |
|  |  | At5g24110 | At5g66650 |
|  |  |  | At2g37430 |
|  |  |  | At3g16720 |
| Metal | At2g35980 | At4g34150 | At1g80440 |
|  | At2g21390 | At5g37690 | At4g34150 |
|  | At1g47400 | At1g47400 | At3g10340 |
|  | At5g23020 | At2g01880 | At1g57990 |
|  |  |  | At1g26850 |
