# Supplementary material for: G-protein Signaling Components GCR1 and GPA1 Mediate Responses to Multiple Abiotic Stresses in Arabidopsis
Source: Front Plant Sci. 2015 Nov 18;6:1000. doi: 10.3389/fpls.2015.01000 (PMC4649046; doi:10.3389/fpls.2015.01000)
Supplement: Supplementary Table S3 — qPCR of stress responsive genes validate the role GPA1 and GCR1 in regulating abiotic stresses. These genes have been implicated in various abiotic stress response previously and also found to be differentially regulated in our transcriptome data (GEO accession no. GSE 40217). The values are given as average of log2 fold change ±SE obtained from 3 independent experiments each having technical triplicates. Values followed by different letters are significantly different at 5% level as determined by Duncan's test. [file Table3.DOCX]

**Supplementary table S3:** **qPCR of stress responsive genes validate the role GPA1 and GCR1 in regulating abiotic stresses.** These genes have been implicated in various abiotic stress response previously and also found to be differentially regulated in our transcriptome data (GEO accession no. GSE 40217). The values are given as average of log2 fold change±SE obtained from 3 independent experiments each having technical triplicates. Values followed by different letters are significantly different at 5 % level as determined by Duncan’s test.

|  | Control | | | | | cold (4 °C) | | | | heat (37 °C) | | | | NaCl (100 mM) | | | |
| --- | --- | --- | --- | --- | --- | --- | --- | --- | --- | --- | --- | --- | --- | --- | --- | --- | --- |
|  | **Ws2** | ***gcr1-5*** | ***gpa1-5*** | ***gpa1-5gcr1-5*** | **Ws2** | | ***gcr1-5*** | ***gpa1-5*** | ***gpa1-5gcr1-5*** | **Ws2** | ***gcr1-5*** | ***gpa1-5*** | ***gpa1-5gcr1-5*** | **Ws2** | ***gcr1-5*** | ***gpa1-5*** | ***gpa1-5gcr1-5*** |
| ERF13 | 0.00±0.00a | 1.60±0.15b | 1.45±0.06c | 1.91±0.12b | 4.23±0.52a | | 5.13±0.32a | 5.93±0.20c | 6.10±0.12bc | -4.87±0.18ab | -2.20±0.06d | -3.24±0.09a | -1.88±0.12e | 9.53±0.35d | 11.90±0.17de | 11.37±0.21bd | 8.93±0.42ab |
| CML37 | 0.00±0.00a | 1.26±0.04b | 1.03±0.10a | 0.78±0.05b | 5.17±0.20bc | | 6.29±0.32b | 5.33±0.07c | 5.08±0.27bc | 2.23±0.09d | 4.00±0.28e | 3.10±0.21b | 3.65±0.30bd | 7.83±0.20de | 10.81±0.26d | 9.23±0.32e | 8.11±0.11f |
| RRTF1 | 0.00±0.00a | 4.00±0.12b | 0.69±0.04c | 0.51±0.02d | -3.60±0.31e | | 0.09±0.04be | -4.00±0.25d | -2.90±0.15b | -2.98±0.19bc | -1.97±0.28bd | -2.87±0.12f | -3.27±0.09de | 8.25±0.32f | 12.33±0.47f | 9.07±0.37d | 6.93±0.35c |
| LTP4 | 0.00±0.00a | -0.28±0.03b | -1.00±0.12c | -1.49±0.19c | -2.60±0.29d | | -2.67±0.29d | -3.16±0.29c | -4.29±0.08bc | 4.73±0.24bc | 4.23±0.32d | 2.94±0.24d | 2.10±0.12cd | 10.64±0.48e | 10.71±0.26e | 8.61±0.09e | 8.26±0.31e |
| WRKY46 | 0.00±0.00a | 1.06±0.09b | 1.31±0.07c | 1.09±0.06b | 3.87±0.22d | | 4.97±0.28e | 4.10±0.10e | 4.50±0.06d | -1.77±0.18bc | -1.73±0.23e | -2.47±0.17f | -3.03±0.22ef | 5.67±0.23e | 6.07±0.19de | 6.90±0.15de | 5.03±0.22g |
| RD26 | 0.00±0.00a | 0.74±0.05a | 0.43±0.04a | 1.15±0.08b | 6.47±0.28c | | 7.03±0.43d | 7.37±0.56c | 8.07±0.09cd | 2.70±0.25b | 3.97±0.28e | 3.97±0.28e | 3.13±0.18de | 5.10±0.26c | 4.90±0.25e | 6.07±0.23c | 5.93±0.27bc |
| ERF6 | 0.00±0.00a | 0.68±0.03a | 0.70±0.04a | 1.12±0.04b | 8.80±0.21c | | 9.07±0.24d | 8.10±0.17c | 11.47±0.35e | -6.73±0.24cd | -6.95±0.21cd | -5.57±0.27bc | -5.90±0.35bc | 5.40±0.21bc | 5.87±0.28bc | 5.97±0.28f | 6.00±0.15g |
| KCS2 | 0.00±0.00a | -0.31±0.02a | -0.44±0.07a | -1.10±0.07b | -3.43±0.19c | | -4.13±0.18bc | -2.41±0.21b | -4.27±0.28bc | 1.57±0.12b | 2.53±0.18d | 1.47±0.18b | 2.20±0.06b | 5.80±0.23bc | 6.07±0.19bc | 5.90±0.25b | 4.40±0.12c |
| CRK11 | 0.00±0.00a | 1.37±0.13b | 0.70±0.08a | 2.01±0.21c | -1.41±0.13d | | 0.15±0.01a | -2.18±0.06e | -2.16±0.43e | -1.58±0.26e | 0.39±0.02a | -2.73±0.19e | -1.85±0.17de | 5.14±0.21f | 9.81±0.10g | 5.74±0.28f | 7.27±0.30fg |
| YLS9 | 0.00±0.00a | -0.57±0.03a | -1.20±0.07b | -0.89±0.05a | -2.97±0.35c | | -3.87±0.18c | -4.53±0.18d | -4.57±0.15d | -2.10±0.17bc | -3.07±0.15c | -3.17±0.09bc | -4.10±0.32d | 5.07±0.39e | 4.83±0.20f | 5.30±0.29e | 3.85±0.19ef |
| RD29A | 0.00±0.00a | 0.44±0.03a | 0.48±0.03a | 1.11±0.06b | 7.67±0.29c | | 7.49±0.32c | 7.28±0.45bc | 8.61±0.27bc | 2.23±0.16b | 2.78±0.23b | 2.84±0.20bc | 5.54±0.30c | 5.89±0.18d | 5.14±0.31cd | 5.15±0.20d | 6.87±0.31e |
| AT-PP2A5 | 0.00±0.00a | 1.92±0.11a | 1.13±0.06b | 2.04±0.16a | 7.73±0.33c | | 9.51±0.29bc | 8.90±0.34d | 10.07±0.11bd | -3.80±0.23e | -1.99±0.16f | -1.31±0.09a | -1.79±0.28ef | 2.61±0.32a | 4.19±0.24ab | 5.06±0.17c | 5.97±0.38cd |
| LOX4 | 0.00±0.00a | 1.33±0.06b | 0.58±0.03a | 1.57±0.09b | 5.10±0.12c | | 7.03±0.12bc | 5.47±0.18c | 5.97±0.28c | -2.20±0.15d | -2.90±0.25d | -2.63±0.28bd | -2.97±0.19 | 6.90±0.26bc | 7.93±0.24c | 5.03±0.22b | 9.83±0.14bd |
| ELIP1 | 0.00±0.00a | 0.09±0.04a | -0.44±0.06a | -1.03±0.04b | 4.37±0.15c | | 4.20±0.06c | 2.10±0.12d | 2.87±0.22d | 3.63±0.30cd | 3.63±0.30d | 2.97±0.28c | 2.73±0.29cd | 4.73±0.62c | 4.93±0.17c | 5.00±0.42cd | 4.40±0.12d |
| CML38 | 0.00±0.00a | 1.29±0.04b | 1.35±0.06b | 1.65±0.07c | 7.53±0.48d | | 7.89±0.39d | 7.92±0.17c | 9.26±0.34bd | -4.36±0.42e | -4.23±0.32f | -2.68±0.28ef | -2.04±0.41e | 1.89±0.21b | 3.67±0.29d | 4.17±0.23bd | 1.65±0.07bc |
| LDOX | 0.00±0.00a | -0.43±0.06a | -2.65±0.24b | -1.95±0.08b | -2.63±0.38b | | -3.53±0.18c | -5.03±0.19d | -4.19±0.22bc | -4.70±0.26bc | -5.43±0.19d | -5.94±0.24b | -4.09±0.12bd | 3.07±0.20e | 1.89±0.14f | 4.03±0.33g | 4.11±0.25g |
| CNI1 | 0.00±0.00a | 1.26±0.11b | 0.71±0.08a | 1.25±0.12b | 1.90±0.21b | | 2.45±0.10c | 2.92±0.24c | 4.15±0.26bc | 2.06±0.18c | 3.87±0.38bc | 1.77±0.26d | 5.97±0.31cd | 3.30±0.15cd | 4.09±0.33d | 3.79±0.24d | 5.29±0.22cd |
| At1g55450 | 0.00±0.00 | 1.24± 0.06b | 0.65± 0.03a | 1.29± 0.01b | 3.03± 0.27c | | 3.93± 0.37c | 3.13± 0.03d | 5.07± 0.19bc | -2.50± 0.40e | -4.87± 0.34f | -2.93± 0.23e | -2.83± 0.19 | 3.67± 0.29bd | 5.07± 0.19bd | 3.70± 0.26d | 6.10± 0.25c |
| ZAT11 | 0.00±0.00a | 2.22± 0.20b | 1.33± 0.04c | 2.31± 0.06b | 2.40± 0.32b | | 5.20± 0.26bc | 3.93± 0.27d | 5.07± 0.19bd | -3.10± 0.20e | -1.60± 0.35f | -3.90± 0.25d | -2.07± 0.19g | 6.43± 0.46d | 8.23± 0.26bd | 3.60± 0.12b | 6.87± 0.18bc |
| CMPG1 | 0.00±0.00a | 1.39± 0.11b | 0.82± 0.06a | 0.70± 0.03a | 3.84± 0.26c | | 5.09± 0.11d | 5.10± 0.06d | 4.13± 0.03c | -1.52± 0.14e | -2.27± 0.09f | -2.17± 0.03f | -2.73± 0.20ef | 2.93± 0.24c | 5.79± 0.22cd | 3.07± 0.14d | 3.07± 0.24d |
| MLO12 | 0.00±0.00a | 2.21± 0.06b | 0.89± 0.05a | 1.10± 0.06b | 1.50± 0.05c | | 4.37± 0.12d | 1.83± 0.15b | 2.35± 0.04b | 1.15± 0.05c | 5.53± 0.28cd | 1.43± 0.12bc | 1.63± 0.12bc | 3.75± 0.16d | 6.97± 0.28e | 2.90± 0.25b | 3.17± 0.28cd |
| VSP2 | 0.00±0.00a | -0.02± 0.01a | -1.19± 0.21b | -1.17± 0.05b | -2.03± 0.15c | | -2.10± 0.21c | -3.03± 0.23d | -3.03± 0.32d | -2.37± 0.29 | -2.40± 0.38c | -3.33± 0.22cd | -3.03± 0.18bd | 2.17± 0.19e | 2.10± 0.21e | 1.73± 0.20f | 1.97± 0.19e |
| NRT2 | 0.00±0.00a | 1.07± 0.08b | 2.00± 0.07b | 0.16± 0.01a | -2.87± 0.24c | | -3.89± 0.17d | -2.14± 0.09c | -2.97± 0.34c | -5.13± 0.58e | -5.06± 0.43e | -2.99± 0.17cd | -4.74± 0.29d | -3.00± 0.38cd | -4.02± 0.20bd | 0.15± 0.02a | -2.88± 0.29e |
| SPX1 | 0.00±0.00a | -1.19± 0.10b | -0.54± 0.03a | -0.42± 0.04a | 3.23± 0.20c | | 2.03± 0.23d | 2.77± 0.23d | 2.90± 0.21cd | -2.10± 0.21ab | -2.77± 0.23b | -2.70± 0.21bc | -2.83± 0.15b | -1.10± 0.15e | -2.37± 0.18be | -1.60± 0.12e | -1.70± 0.15be |
| PDF1.2 | 0.00±0.00a | 1.53± 0.05b | 0.37± 0.03a | -0.56± 0.03c | -1.73± 0.28d | | -3.93± 0.32e | -1.87± 0.12d | -2.07± 0.15bd | -3.87± 0.29de | -5.80± 0.23f | -4.13± 0.20ef | -4.07± 0.19f | -2.13± 0.26d | -3.67± 0.29f | -2.09± 0.12d | -2.37± 0.15d |
| ASN1 | 0.00±0.00a | -0.72± 0.05a | 1.24± 0.05b | 1.79± 0.03b | -3.67± 0.29c | | -4.20± 0.06d | -2.09± 0.10e | -4.17± 0.26d | 2.47± 0.18b | 3.00± 0.20c | 2.27± 0.19bc | 3.90± 0.25e | -2.43± 0.27c | -2.63± 0.23cd | -3.37± 0.22d | -3.07± 0.20f |
| At4g30170 | 0.00±0.00a | 0.14± 0.03a | 1.59± 0.03b | 0.88± 0.05c | -2.73± 0.20d | | -2.70± 0.21d | -2.80± 0.35d | -2.77± 0.18d | -3.20± 0.25e | -3.17± 0.38de | -4.33± 0.33e | -3.07± 0.26d | -2.83± 0.23c | -3.13± 0.26b | -3.87± 0.23de | -3.03± 0.18bd |
| At5g19890 | 0.00±0.00a | 0.83± 0.05a | 0.77± 0.06a | 1.19± 0.10b | -1.50± 0.06c | | -2.23± 0.09d | -2.00± 0.15d | -2.33± 0.09c | -1.53± 0.30c | -2.37± 0.12cd | -1.50± 0.15d | -2.53± 0.13d | -4.50± 0.36e | -4.53± 0.18f | -4.97± 0.28g | -4.97± 0.19g |
